# *Flash Communication:* Challenges in the Characterization of a Bisphosphine Nickel Fluoride Complex

**DOI:** 10.1021/acs.organomet.6c00218

**Published:** 2026-09-01

**Authors:** Josie B. Rojo, Aurora F. Wilson, David A. Vicic

**Affiliations:** † Department of Chemistry, 1687 Lehigh University, 6 E Packer Ave., Bethlehem, Pennsylvania 18015, United States; ‡ Department of Chemistry, University of the Philippines Visayas, Miagao, Iloilo 5023, Philippines

## Abstract

A prior report (*Inorg. Chim. Acta* **2011**, 376, 118–122) detailed the use of “[Ni­(THF)_6_]­[(BF_4_)_2_]” to prepare [(dtbe)­Ni­(μ-F)]_2_[BF_4_]_2_ (dtbe = 1,2-bis­(di-*tert*-butylphosphino)­ethane) using a DCM/THF (1:1) solvent system. Here we describe a procedure that affords [Ni­(THF)_4_(BF_4_)_2_] in the solid-state, and note that reaction of [Ni­(THF)_4_(BF_4_)_2_] with the similar phosphine dcpe (dcpe = 1,2-bis­(dicyclohexylphosphino)­ethane) only produces [(dcpe)­Ni­(μ-F)]_2_[BF_4_]_2_ when run in thoroughly dried THF solvent. If a 1:1 mixture of DCM/THF solvents is used as previously described, the bridging chloride derivative [(dcpe)­Ni­(μ-Cl)]_2_[BF_4_]_2_ is formed. Moreover, we show that bridging fluorides of [(dcpe)­Ni­(μ-F)]_2_[BF_4_]_2_ can be short-lived in solution, reacting with the medium to produce unexpected products.

## Graphical abstract

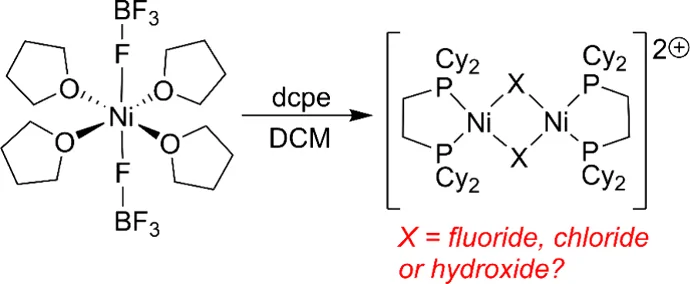

It was reported that the nickel precursor “[Ni­(THF)_6_(BF_4_)_2_]” (**1**) could be prepared from the dehydration of [Ni­(H_2_O)_6_]­[(BF_4_)_2_] in THF solvent.^1^ We discovered that, with the procedure outlined herein, the isolated solid produced is not **1** but rather compound **2** where the axial THF ligands are replaced by coordinated [BF_4_] counterions (Scheme 1). This formulation of the isolated solid is important to establish if the nickel complex is indeed to be properly used as a precursor to other coordination compounds of nickel. The structure of **2** was determined by X-ray diffractometry and elemental analysis, and the ORTEP diagram is shown in Figure 1. Of note is the weakened B–F bond of the fluorine that is coordinated to nickel versus those that are not (1.440(5) Å vs an average of 1.363(9) Å). The axial placement of the [BF_4_] groups is reminiscent of the related [Ni­(THF)_4_(OTf)_2_] (**3**) derivative.^2^

**Scheme 1:**
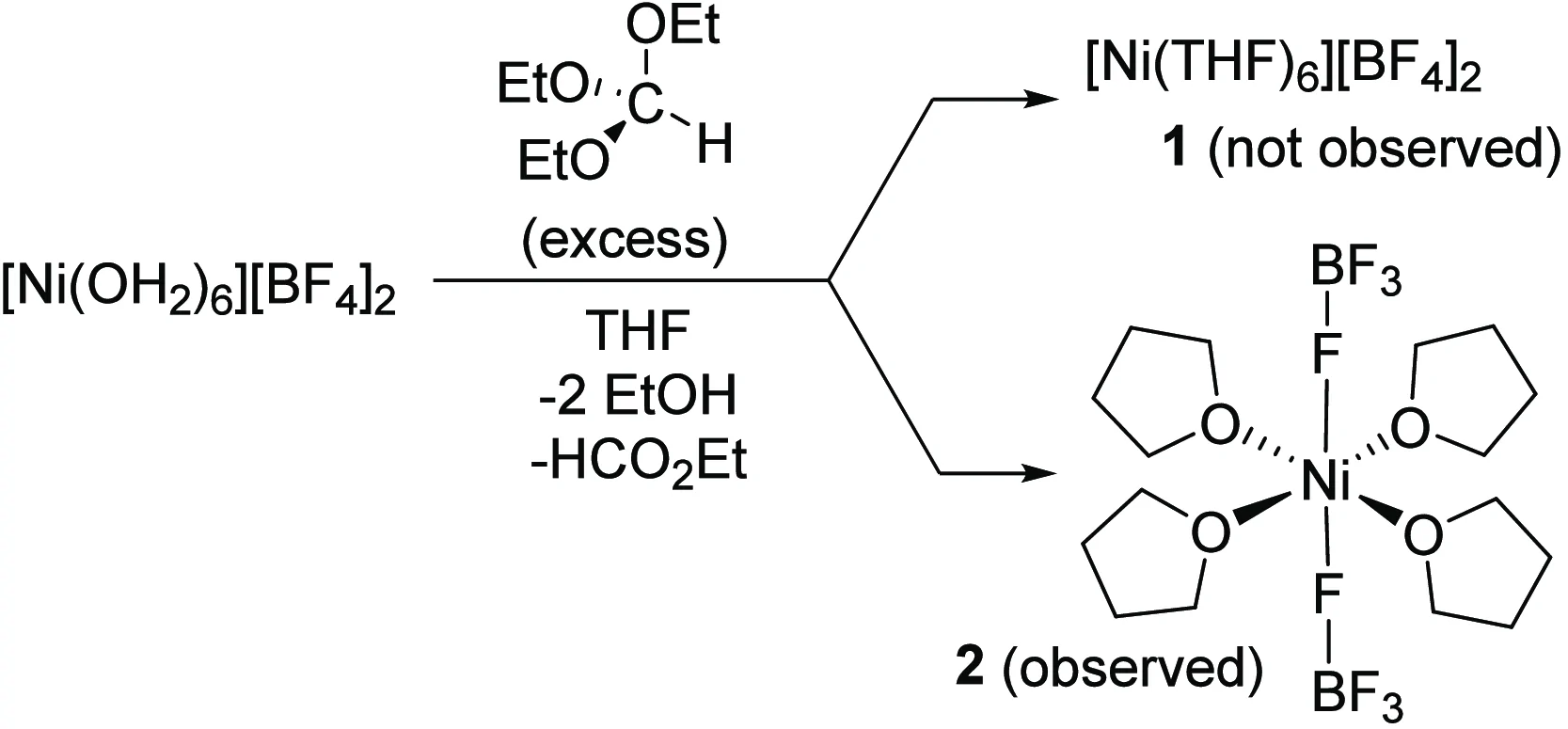
Product Observed in the Solid-State upon Dehydration of [Ni­(H_2_O)_6_]­[BF_4_]_2_

**Figure 1:**
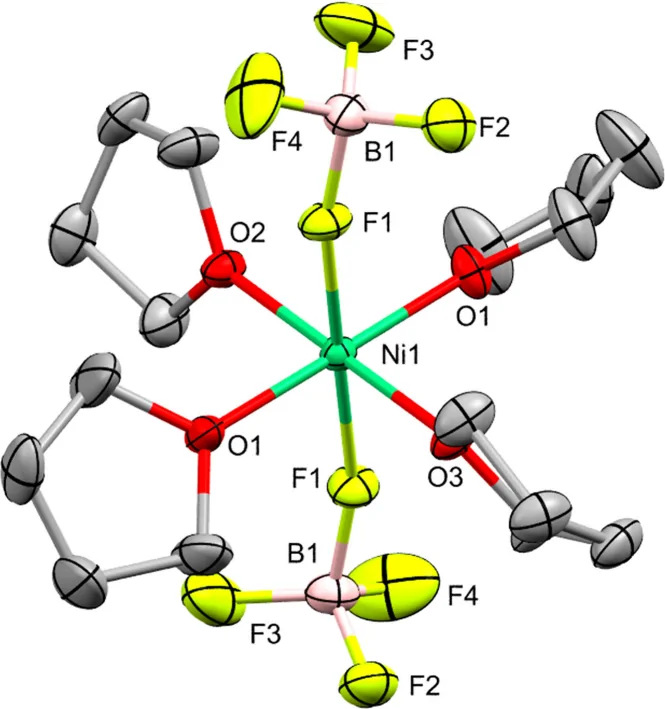
ORTEP diagram of **2**. Hydrogen atoms are omitted for clarity. Thermal ellipsoids are shown at the 50% level. Selected bond lengths (Å): Ni1–O1 2.007(2); Ni1–O1 2.007(2); Ni1–O3 2.032(3); Ni1–O2 2.039(3); Ni1–F1 2.0603(18); F1–B1 1.440(5); F2–B1 1.365(5). Selected bond angles (deg): O1–Ni1–O1 179.52(14); O1–Ni1–O3 90.24(7); O1–Ni1–O3 90.24(7); O1–Ni1–O2 89.76(7); O1–Ni1–O2 89.76(7); O3–Ni1–O2 180.0; O1–Ni1–F1 91.28(9); O1–Ni1–F1 88.72(9); O3–Ni1–F1 89.60(6); O2–Ni1–F1 90.40(6); O1–Ni1–F1 88.72(9); O1–Ni1–F1 91.28(9); O3–Ni1–F1 89.60(6); O2–Ni1–F1 90.40(6); F1–Ni1–F1 179.19(11); B1–F1–Ni1 161.5(2).

That same prior report^1^ also detailed how reaction of precursor **1** with dtbe in dichloromethane/THF mixtures led to the formation of [(dtbe)­Ni­(μ-F)]_2_[BF_4_]_2_ through decomposition of the [BF_4_] counterions. The authors reported two signals in the ^19^F NMR spectrum for [(dtbe)­Ni­(μ-F)]_2_[BF_4_]_2_. The first, which integrated to two fluorines, was attributed to the bridging fluoride ligand at δ = −152.1 in CD_2_Cl_2_ solvent. The second, which integrated to eight fluorines, was attributed to the [BF_4_] groups and reportedly appeared at δ = −152.2. However, in our experiences, two resonances that appear in this region are typically *both* attributable to the [BF_4_] counterion stemming from ^10^B and ^11^B having 2:8 relative isotopic abundances, respectively. Since bridging fluorides of nickel appear in ranges upfield of δ = −300 in the ^19^F NMR window,^3,4^ we investigated the coordination of a chelating phosphine ligand with the newly formulated precursor **2** further.

In our hands, reaction of **2** or **3** with dcpe in the previously reported THF:CH_2_Cl_2_ (1:1) solvent system did not give a bridging fluoride compound but instead gave the bridging chloride dimers **4a** and **4b** (eq 1). Compounds **4a** and **4b** each display a singlet in the ^31^P­{^1^H} NMR spectrum at δ = 95.3 and 95.4, respectively, in CD_2_Cl_2_. Compound **4a** was structurally characterized, and select crystallographic data are provided in the Supporting Information.
1

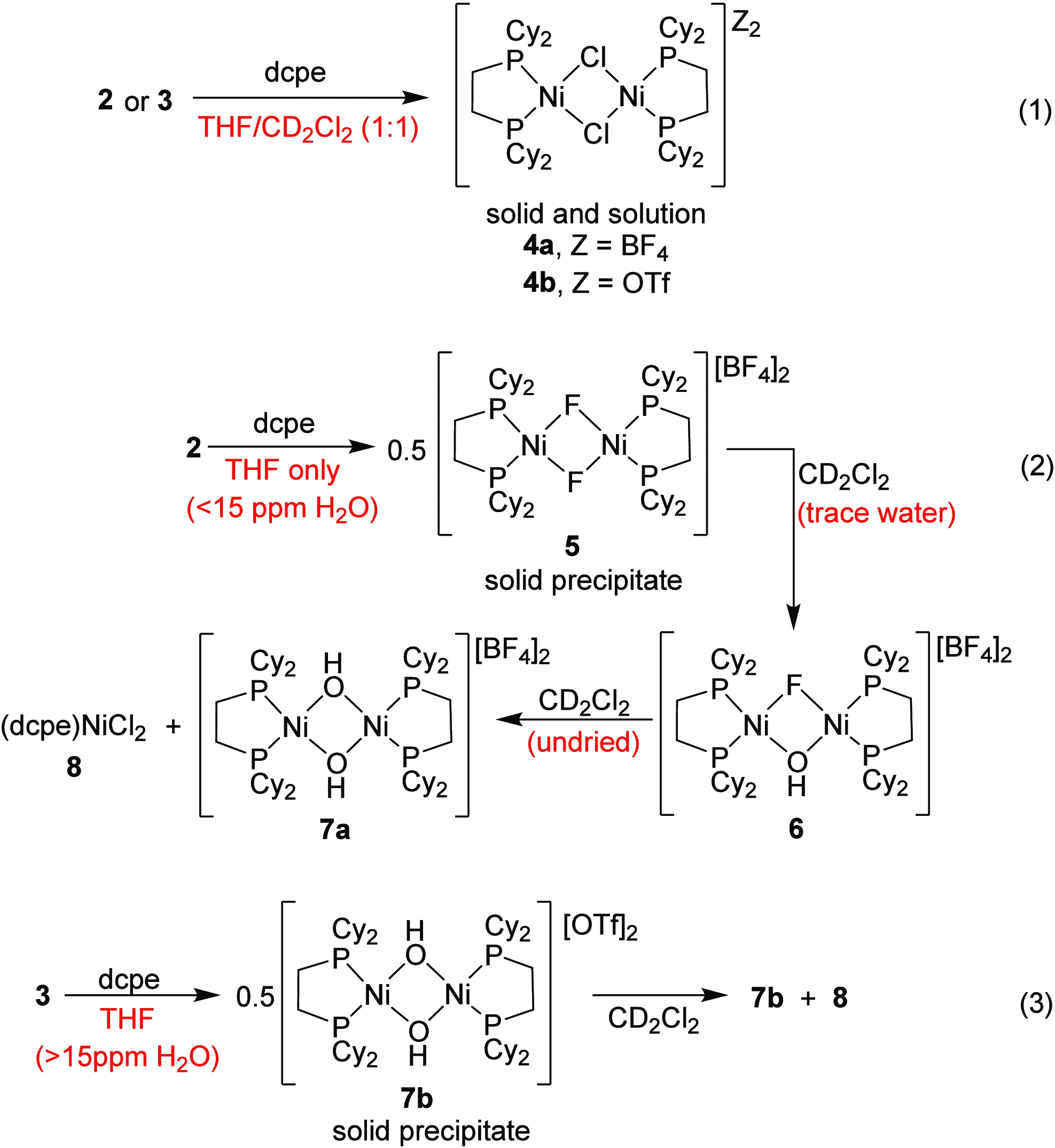




The bridging fluoride dimer **5** was only formed in reasonable amounts when the addition of dcpe was performed in thoroughly dried and nonhalogenated solvent like THF (eq 2) in a fluoropolymer PFA vessel. The analysis of **5** was not straightforward, as it is insoluble in nonhalogenated solvents. In dichloromethane, the ^31^P­{^1^H} NMR spectrum of **5** resembled that of [(dtbe)­Ni­(μ-F)]_2_[BF_4_]_2_, giving a second-order signal centered at δ = 89.0.^1^ The ^19^F NMR spectrum showed a signal for the bridging fluoride at δ = −411.0 in dichloromethane, which confirms that the [BF_4_] counterion was previously misassigned as the bridging fluorides in [(dtbe)­Ni­(μ-F)]_2_[BF_4_]_2_.^1^ The ^19^F NMR resonances of **5** were challenging to record, as they required dried (<0.7 ppm of H_2_O) CD_2_Cl_2_ and the use of a PFA liner in the NMR tube. Despite this care, the conversion of **5** to **6** in the NMR tube could be observed by NMR spectroscopy (eq 2). In the ^31^P­{^1^H} NMR spectrum, compound **6** displays two doublet of doublets appearing at δ = 89.3 (*J* = 108.5 and 62.4 Hz) and δ = 82.0 (*J* = 62.7 and 22.5 Hz) indicating a less symmetric structure in solution. In the ^19^F­{^1^H} NMR spectrum, the bridging fluoride of **6** appears as a tt (*J*_P(trans)‑F_ = 107.4 and *J*_(Pcis)‑F_ = 22.6 Hz) at δ = – 447.5 in CD_2_Cl_2_. This signal for the bridging fluoride of **6** broadens when the ^19^F channel is not proton decoupled, highlighting the coupling of the fluoride to the OH moiety as also seen in the ^1^H NMR spectrum (see Supporting Information). If undried CD_2_Cl_2_ is used as a solvent in a glass NMR tube, conversion of **5** to the fully defluorinated **7a** was observed (eq 2). Compound **7a** is symmetric and gives a singlet in the ^31^P­{^1^H} NMR (δ = 82.4). Both **6** and **7a** were further characterized by their diagnostic O–H resonances in their ^1^H NMR spectra,^5^ which appear upfield from zero, with **6** displaying a larger *J*_OH‑F_ (*J* = 10.7 Hz) component at δ = – 0.2 and **7a** displaying only the smaller *J*_OH‑P_ (*J* = 2.6 Hz) at δ = – 2.0 in CD_2_Cl_2_. Finally, we report herein that the hydroxide dimer **7b**, bearing the triflate counterions, is produced upon the addition of dcpe to precursor **3** as described in eq 3. No evidence of any defluorination of triflate is observed, in contrast to [BF_4_]. The reaction in eq 3 is akin to the known preparation of [(dippe)­Ni­(μ–OH)]_2_[OTf]_2_ where [(dippe)­NiCl_2_] (dippe = 1,2-bis­(di-*i*-propylphosphino)­ethane) is reacted with AgOTf followed by crystallization from wet acetone.^5^ Some [(dcpe)­NiCl_2_] (**8**) is observed in the reactions described in eqs 2 and 3, and the similarity of the ^31^P­{^1^H} NMR resonances of **8** in CD_2_Cl_2_ (δ = 82.4) to **7b** (δ = 83.4) demands caution when making assignments. This is especially true for the reaction in eq 2, where the singlet for **8** cannot be resolved from that of **7a** and appears as a shoulder of the resonance for **7a** in the ^31^P­{^1^H} NMR spectrum.

In summary, since nickel phosphine complexes bearing bridging fluorides are rare (only two prior reports),^1,4^ proper characterization of these unusual species is needed to develop a better understanding of their reactivities. This is especially true because crystallographically determined bond lengths for the Ni–F bonds in [LNi­(μ-F)]_2_ complexes are very similar to the Ni–OH bond lengths in [LNi­(μ-OH)]_2_,^1,5^ and one can easily misidentify one for another based on X-ray data alone given the proximity of fluorine to oxygen on the periodic table. Furthermore, standard C,H-elemental analyses cannot reliably distinguish a fluoride derivative from a hydroxide one in compounds such as **5** and **7a**. These compounding issues are rendered even more problematic because, as we have shown here, nickel-fluorides can easily be converted into their nickel-hydroxide counterparts by reaction with the media.

## Supplementary Material



## Notes

Experimental procedures and NMR spectral data (PDF)
