# Supplementary material for: Flash Communication: Challenges in the Characterization of a Bisphosphine Nickel Fluoride Complex
Source: Organometallics. 2026 Sep 1;45(17):2088–90. doi: 10.1021/acs.organomet.6c00218 (PMC13580180; doi:10.1021/acs.organomet.6c00218)
Supplement: Supplementary file 1 [file om6c00218_si_001.pdf]

Supporting Information for

Flash Communication: Challenges in the Characterization of a Bisphosphine Nickel Fluoride Complex

Josie B. Rojo,[†,‡] Aurora F. Wilson,[†] and David A. Vicic[†*]

[†]Department of Chemistry, Lehigh University, 6 E Packer Ave., Bethlehem, PA 18015, USA.

[‡]Department of Chemistry, University of the Philippines Visayas, Miagao, Iloilo 5023, Philippines.

Table of Contents

Page S2: General considerations

Pages S2-S4: Experimental procedures

Pages S5-S36: NMR spectral data

Page S37: ORTEP diagram of [(dcpe)Ni(μ-Cl)]$_2$[BF$_4$]$_2$ (4a)

Page S38: References

General Considerations. All manipulations were performed using standard Schlenk and high vacuum techniques or in an inert gas-filled glovebox (N_2 or Ar), unless otherwise stated. Solvents were purified by passing through activated alumina and/or copper in a solvent purification system supplied by Pure Process Technology. ^{1}H NMR spectra were recorded at ambient temperature on a Bruker 400 MHz spectrometer and referenced to residual proton solvent signals. ^{19}F NMR spectra were recorded on a Bruker NMR spectrometer operating at 376 MHz and referenced to α,α,α-trifluorotoluene (TFT) as an internal standard (δ = −63.72). A Bruker D8 Quest diffractometer was used for X-ray crystal structure determinations. Water content was determined vis use of a Mettler Toledo C20 Coulometric Karl Fischer titrator.

Preparation of [(THF)$_4$Ni(BF$_4$)$_2$] (2): In a glovebox, triethyl orthoformate (4.2 mL, 25.3 mmol) was added into a solution of $[Ni(H_2O)_6][BF_4]_2$ (1.11 g, 3.26 mmol) in 2.5 mL of THF. The green mixture was stirred at 60 °C for 24 h. The solvent, excess starting material and by-products were removed by high vacuum at 60 °C to obtain a yellow-green solid containing some X-ray quality crystals (1.48 g, 87%). Anal. Calcd (found) for $C_{16}H_{32}B_2F_8NiO_4$: C, 36.91 (36.98); H, 6.19 (6.13).

Alternate preparation of the known[1] [(THF)$_4$Ni(OTf)$_2$] (3): In a glovebox, (1,2-dimethoxyethane)nickel dibromide ([NiBr$_2$(dme)]) (0.80 g, 2.60 mmol) was added into a solution of silver triflate (AgOTf) (1.30 g, 5.06 mmol) in 15 mL of THF. The mixture was stirred at room temperature for 30 min and filtered. The filter cake was washed by THF three times (3 x 10 mL). The THF washings were combined with the filtrate. The solvent was then pumped off by high vacuum to obtain a yellow green powder (1.35 g, 83%). X-ray quality crystals were obtained by vapor diffusion with pentane of a saturated solution of the powder in THF.

Preparation of [(dcpe)Ni(μ-Cl)]$_2$[BF$_4$]$_2$ (4a): In a glovebox, 1,2-bis(dicyclohexylphosphanyl)ethane (dcpe) (8.6 mg, 0.020 mmol) was added into a solution of (THF)$_4$Ni(BF$_4$)$_2$ (10.6 mg, 0.020 mmol) in 2.2 mL solution containing THF and DCM (1:1 v/v). The yellow solution was heated at 80 °C for 24 h. The solvent was removed by high vacuum and the solids washed with THF (0.1 mL x 3) to obtain X-ray quality crystals (10.5 mg, 87%). ^{1}H NMR (400 MHz, CD_2Cl_2) δ = 2.73 – 2.23 (m, 4H), 2.23 – 0.92 (m, 44H). $^{31}P\{^{1}H\}$ NMR (162 MHz, CD_2Cl_2) δ = 95.32. ^{19}F NMR (376 MHz, CD_2Cl_2) δ = –156.75 (s, $^{10}BF_4$), –156.81 (s, $^{11}BF_4$). Anal. Calcd (found) for $C_{52}H_{96}B_2Cl_2F_8Ni_2P_4$: C, 51.74 (51.55); H, 8.02 (8.07).

Preparation of [(dcpe)Ni(μ-Cl)]$_2$[OTf]$_2$ (4b): In a glovebox, 1,2-bis(dicyclohexylphosphanyl)ethane (dcpe) (4.6 mg, 0.011 mmol) was added into a solution of (THF)$_4$Ni(OTf)$_2$ (7.1 mg, 0.011 mmol) in 1.2 mL solution containing THF and DCM (1:1 v/v). The yellow solution was heated at 80°C for 48 h. The solvent was removed by high vacuum and the solids washed with THF (0.1mL x 3) to obtain an orange solid (6.5 mg, 89%). ^{1}H NMR (400 MHz, CD_2Cl_2) δ 2.57 – 2.27 (m, 4H),

2.17 – 1.11 (m, 44H). $^{31}P\{^1H\}$ NMR (162 MHz, CD_2Cl_2) δ = 95.42. ^{19}F NMR (CD_2Cl_2, 376 MHz): δ = –77.94. Anal. Calcd (found) for $C_{54}H_{96}Cl_2F_6Ni_2O_6P_4S_2$: C, 48.71 (48.48); H, 7.27 (7.20).

Preparation of $[Ni_2(\mu\text{-}F)_2(dcpe)_2][BF_4]_2$ (5): In a glovebox, 1,2-bis(dicyclohexylphosphanyl)ethane (dcpe) (4.0 mg, 0.010 mmol) was added into a solution of $(THF)_4Ni(BF_4)_2$ (4.5 mg, 0.009 mmol) in 1.0 mL solution of dry THF. The yellow suspension was heated at 80 °C for 24 h. The orange crystals precipitated out of the solution. The solvent was removed by careful decantation and the orange solids were then washed with THF (0.1 mL x 3) to obtain X-ray quality crystals (4.6 mg, 71%). Compound **5** was insoluble in: pentane, diethyl ether, THF, benzene, DMF, iodobenzene, chlorobenzene, fluorobenzene, and trifluorotoluene. ^1H NMR (400 MHz, CD_2Cl_2) δ 2.93 – 2.75 (m, 1H), 2.43 – 2.14 (m, 5H), 2.14 – 1.02 (m, 90H). $^{31}P\{^1H\}$ NMR (162 MHz, CD_2Cl_2) δ 90.20 – 88.61 (m). ^{19}F NMR (376 MHz, CD_2Cl_2) δ -150.97, -410.46 – -411.55 (m). Anal. Calcd (found) for $C_{52}H_{96}B_2F_{10}Ni_2P_4$: C, 53.19 (53.04); H, 8.24 (8.02).

Preparation of $[(dcpe)Ni(\mu\text{-}OH)]_2[OTf]_2$ (7b): In a glovebox, 1,2-bis(dicyclohexylphosphanyl)ethane (dcpe) (6.6 mg, 0.016 mmol) was added into a suspension of $[(THF)_4Ni(OTf)_2]$ (6.8 mg, 0.011 mmol) in 1.5 mL of THF. The very pale-yellow suspension was heated at 80 °C for 72 h. The orange crystals precipitated out of the solution and the solvent was removed by careful decantation. Then solids were then washed with THF (0.1mL x 3) to obtain X-ray quality crystals (6.5 mg, 91%). ^1H NMR (400 MHz, CD_2Cl_2) δ = 3.04 – 2.88 (m, 8H), 2.29 – 2.13 (m, 8H), 2.06 – 1.17 (m, 32H), –1.54 (m, 2H). $^{31}P\{^1H\}$ NMR (162 MHz, CD_2Cl_2) δ = 83.39. ^{19}F NMR (376 MHz, CD_2Cl_2) δ = –77.95. Anal. Calcd (found) for $C_{54}H_{98}F_6Ni_2O_8P_4S_2$: C, 50.09 (49.86); H, 7.63 (7.55).

NMR identification of $[(dcpe)_2Ni_2(\mu\text{-}OH)(\mu\text{-}F)][BF_4]_2$ (6): ^1H NMR (400 MHz, CD_2Cl_2) δ 2.95 – 2.67 (m, 4H), 2.39 – 2.28 (m, 4H), 2.28 – 2.12 (m, 4H), 2.12 – 1.15 (m, 36H), -0.20 (d, J = 10.7 Hz, 1H). $^1H\{^{19}F\}$ NMR (400 MHz, CD_2Cl_2) δ -0.16 (s, 2H). $^1H\{^{31}P\}$ NMR (400 MHz, CD_2Cl_2) δ –0.16 (d, J = 11.6 Hz). $^{31}P\{^1H\}$ NMR (162 MHz, CD_2Cl_2) δ 89.29 (dd, J = 108.5, 62.4 Hz), 81.96 (dd, J = 62.7, 22.5 Hz). ^{19}F NMR (376 MHz, CD_2Cl_2) δ -150.48 (s), –447.49 (ttd, J = 109.0, 24.4, 11.3 Hz). $^{19}F\{^1H\}$ NMR (376 MHz, CD_2Cl_2) δ –447.52 (tt, J = 107.4, 22.6 Hz).

NMR identification of $[(dcpe)Ni(\mu\text{-}OH)]_2[BF_4]_2$ (7a): ^1H NMR (400 MHz, CD_2Cl_2) δ 2.95 – 2.67 (m, 4H), 2.39 – 2.28 (m, 4H), 2.28 – 2.12 (m, 4H), 2.12 – 1.15 (m, 36H), –2.04 (q, J = 2.6, 2H). $^{31}P\{^1H\}$ NMR (162 MHz, CD_2Cl_2) δ 82.39. ^{19}F NMR (376 MHz, CD_2Cl_2) δ = –150.48 (s).

Alternate preparation of the known[2] $[(dcpe)NiCl_2]$ (8): In a glovebox, 1,2-bis(dicyclohexylphosphanyl)ethane (dcpe) (60.2 mg, 0.142 mmol) was added into a solution of (1,2-dimethoxyethane)nickel dichloride $[NiCl_2(dme)]$ (31.4 mg, 0.143 mmol) in 10 mL DCM. The orange mixture was stirred for 16 h. The solution was filtered with 0.45 µm syringe filter and the solvent from the filtrate was pumped off by high vacuum. The resulting residue was washed by diethyl ether

(0.5 mL x 3) to recover an orange product (67.9 mg, 87%). ^{1}H NMR (400 MHz, CD_2Cl_2) δ = 2.65 – 2.50 (m, 4H), 2.27 – 2.07 (m, 4H), 1.96 –1.19 (m, 40H). $^{31}P\{^{1}H\}$ NMR (162 MHz, CD_2Cl_2) δ = 82.44.

Figure S1. ^{1}H NMR spectrum of $[(dcpe)Ni(\mu\text{-}Cl)]_2[BF_4]_2$ (**4a**) in CD_2Cl_2

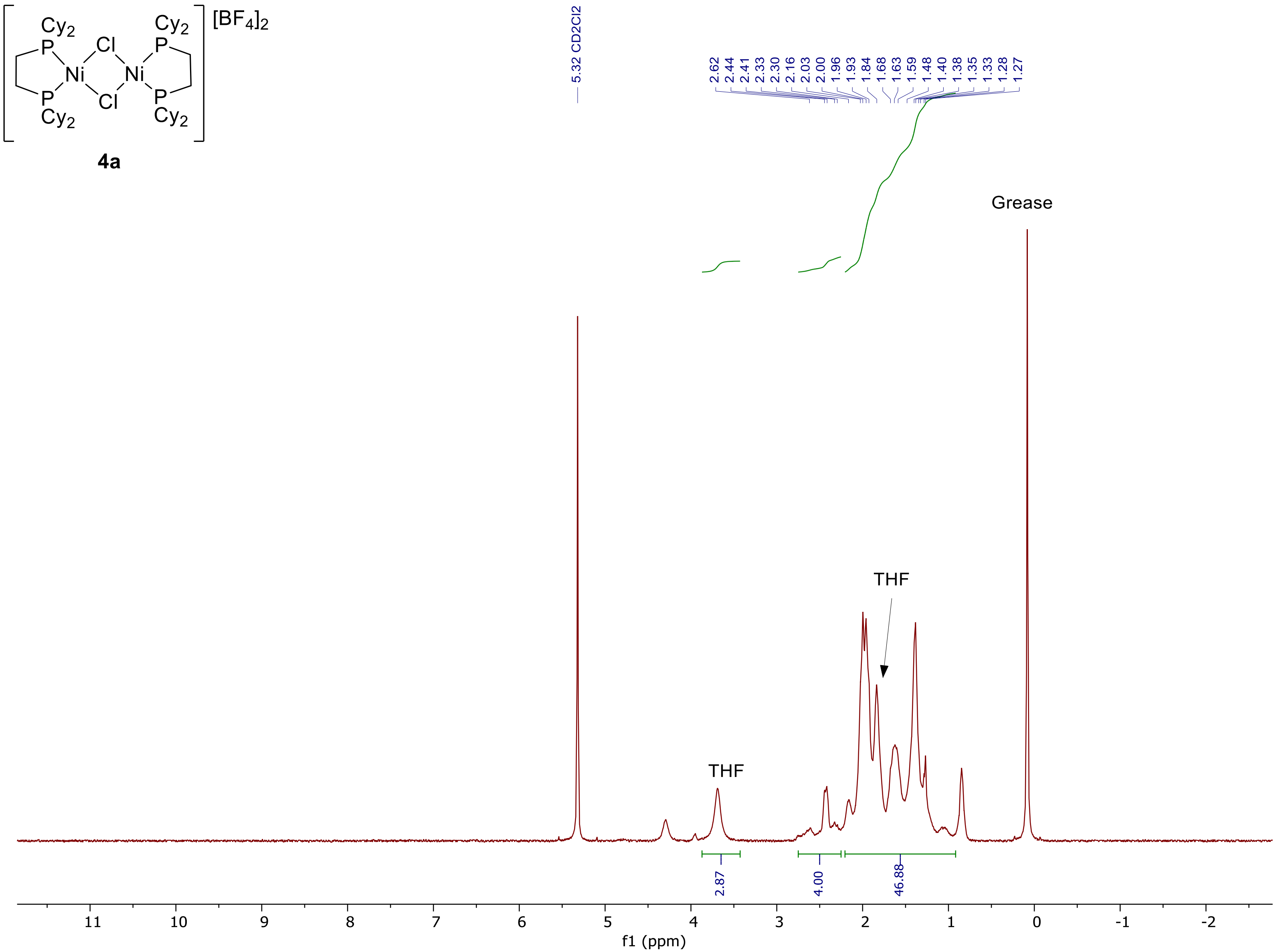

Figure S2. $^{31}P\{^{1}H\}$ NMR spectrum of $[(dcpe)Ni(\mu\text{-}Cl)]_2[BF_4]_2$ (**4a**) in CD_2Cl_2.

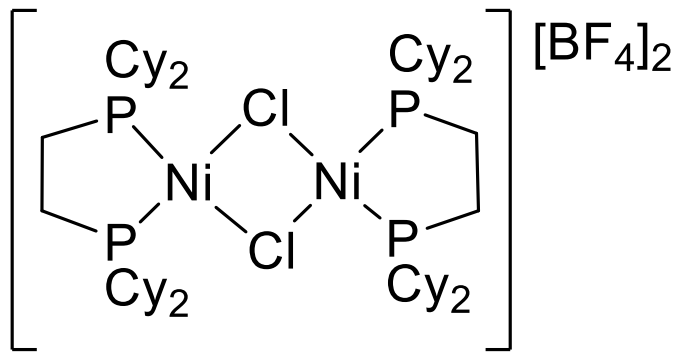

4a

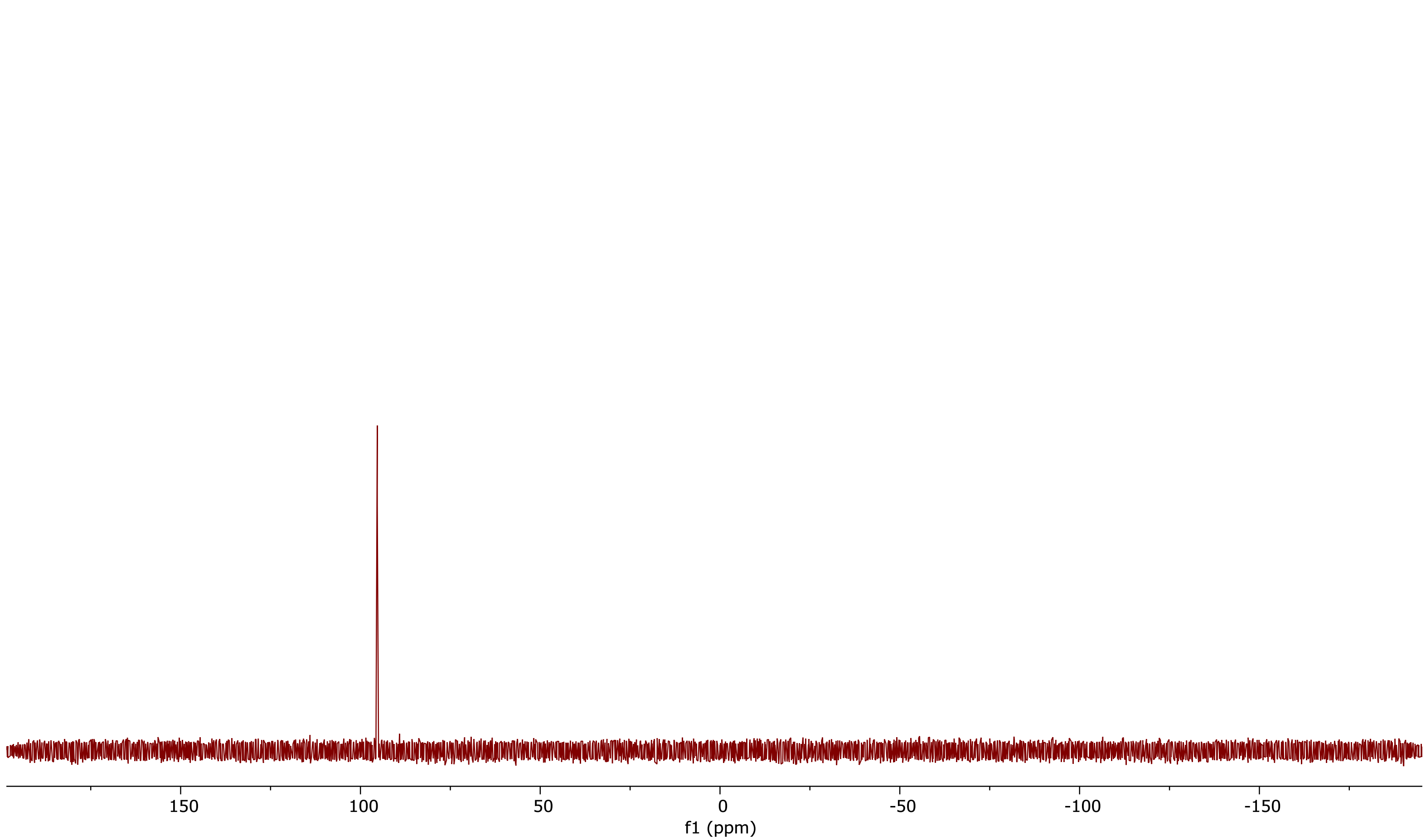

Figure S3. ^{19}F NMR spectrum of $[(dcpe)Ni(\mu\text{-}Cl)]_2[BF_4]_2$ (**4a**) in CD_2Cl_2.

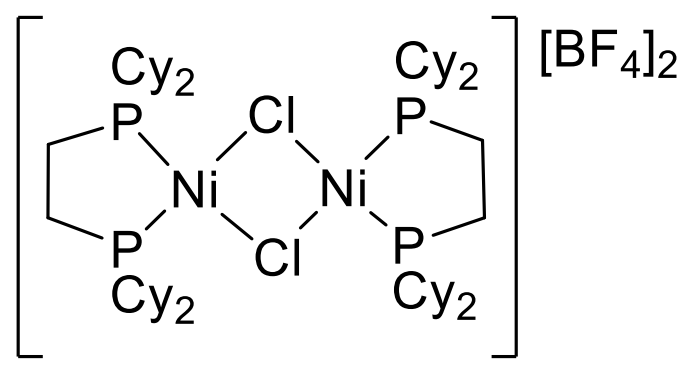

4a

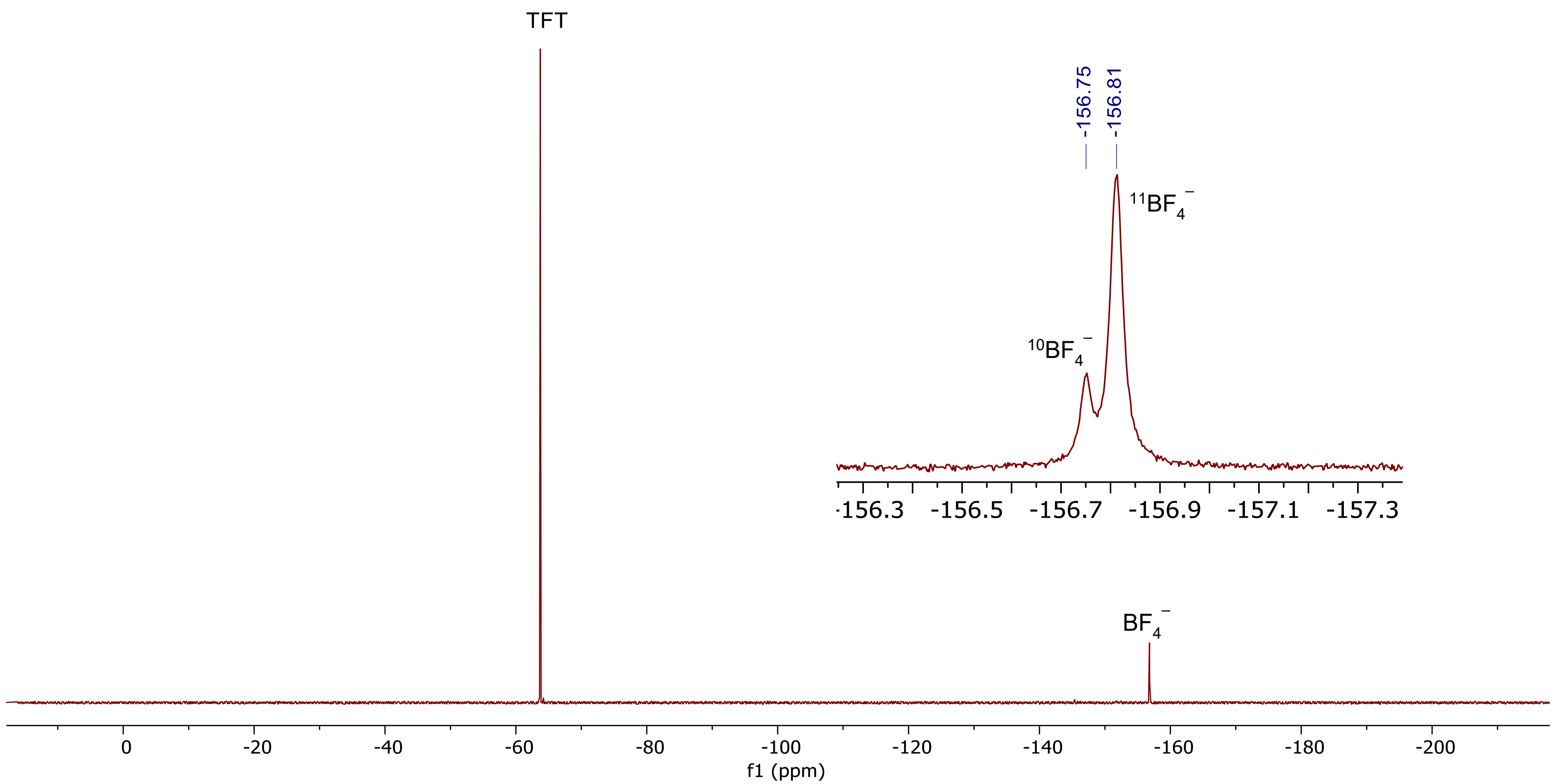

Figure S4. ^{1}H NMR spectrum of [(dcpe)Ni(μ-Cl)]$_2$[OTf]$_2$ (**4b**) in CD_2Cl_2.

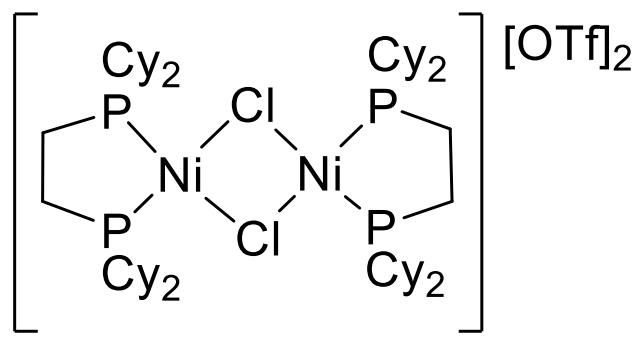

4b

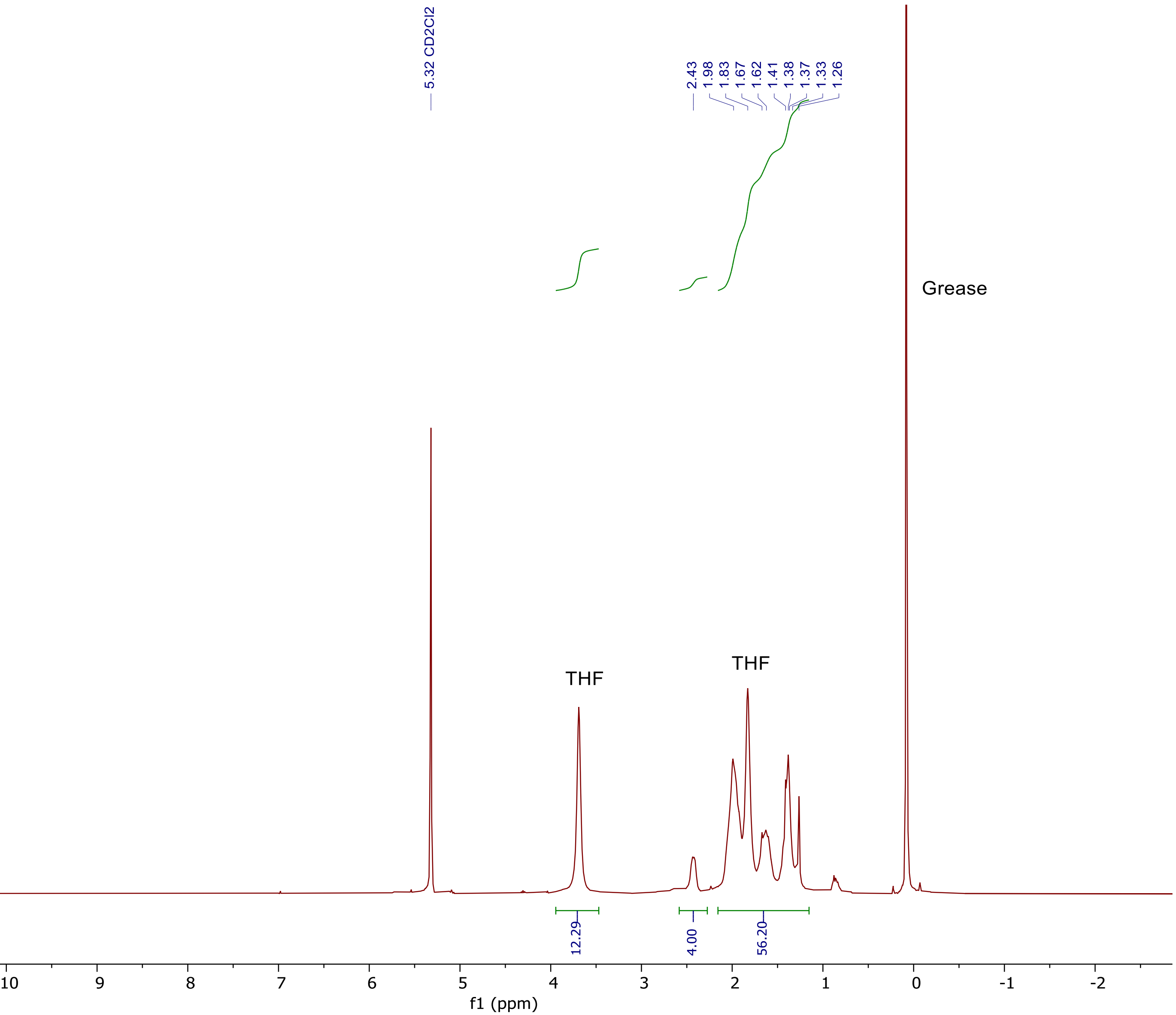

Figure S5. $^{31}P\{^1H\}$ NMR spectrum of $[(dcpe)Ni(\mu\text{-}Cl)]_2[OTf]_2$ (**4b**) in CD_2Cl_2.

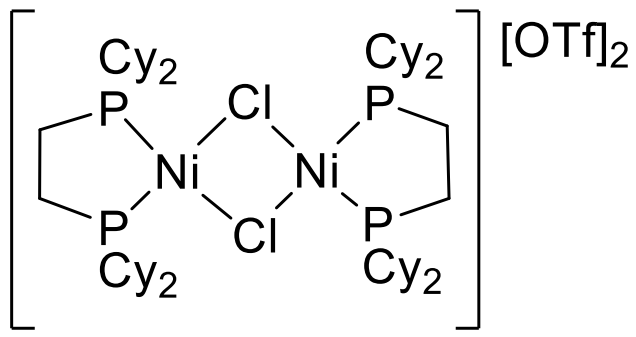

4b

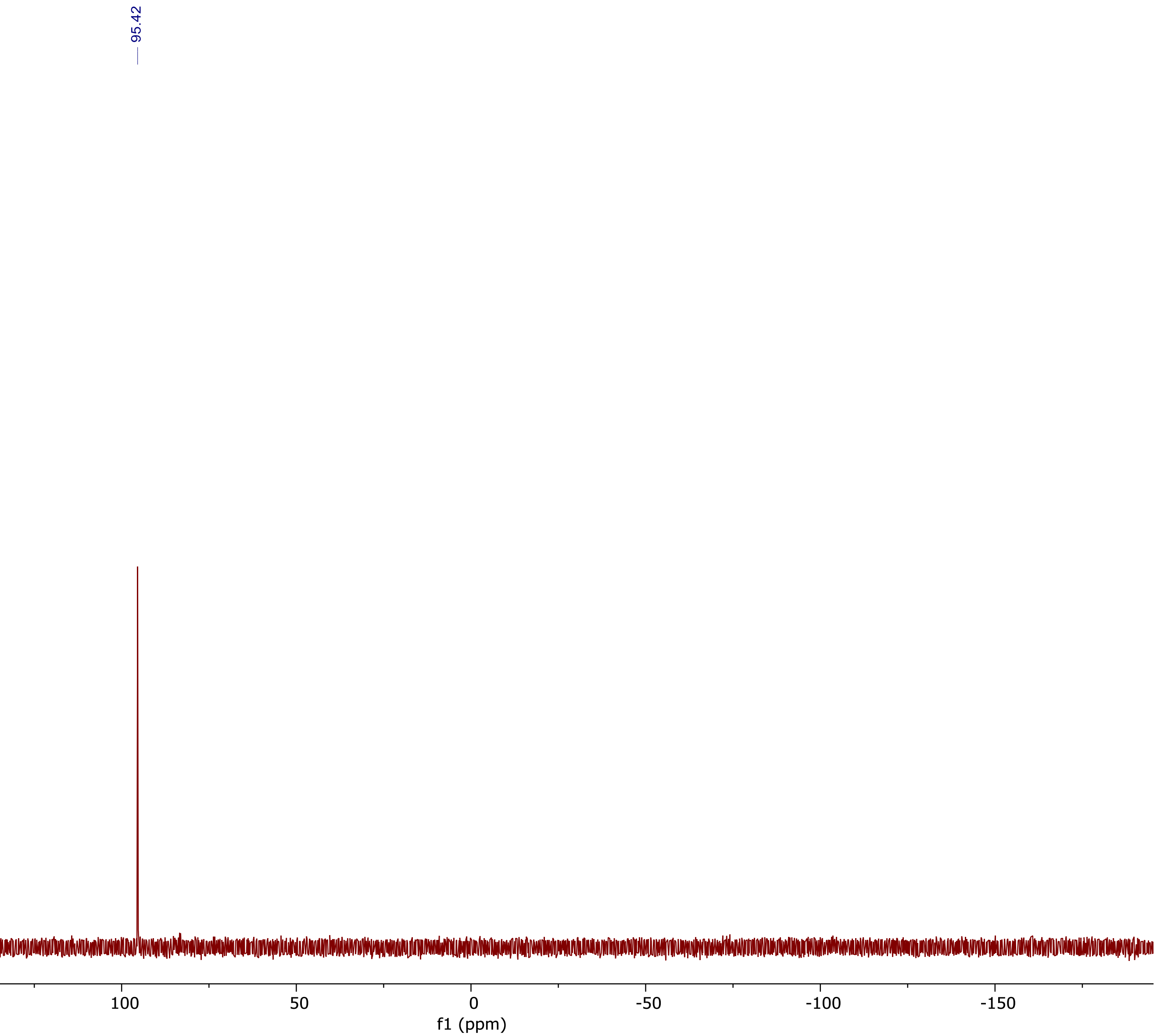

Figure S6. ^{19}F NMR spectrum of $[(dcpe)Ni(\mu\text{-}Cl)]_2[OTf]_2$ (**4b**) in CD_2Cl_2.

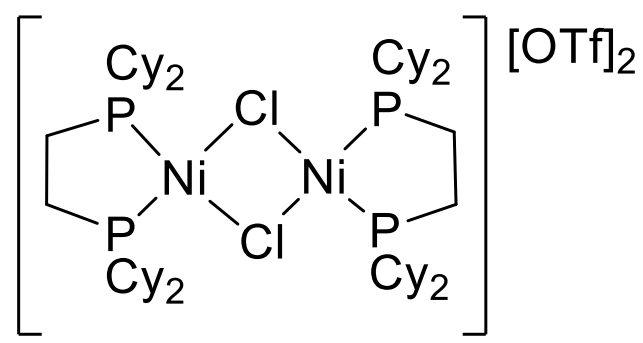

4b

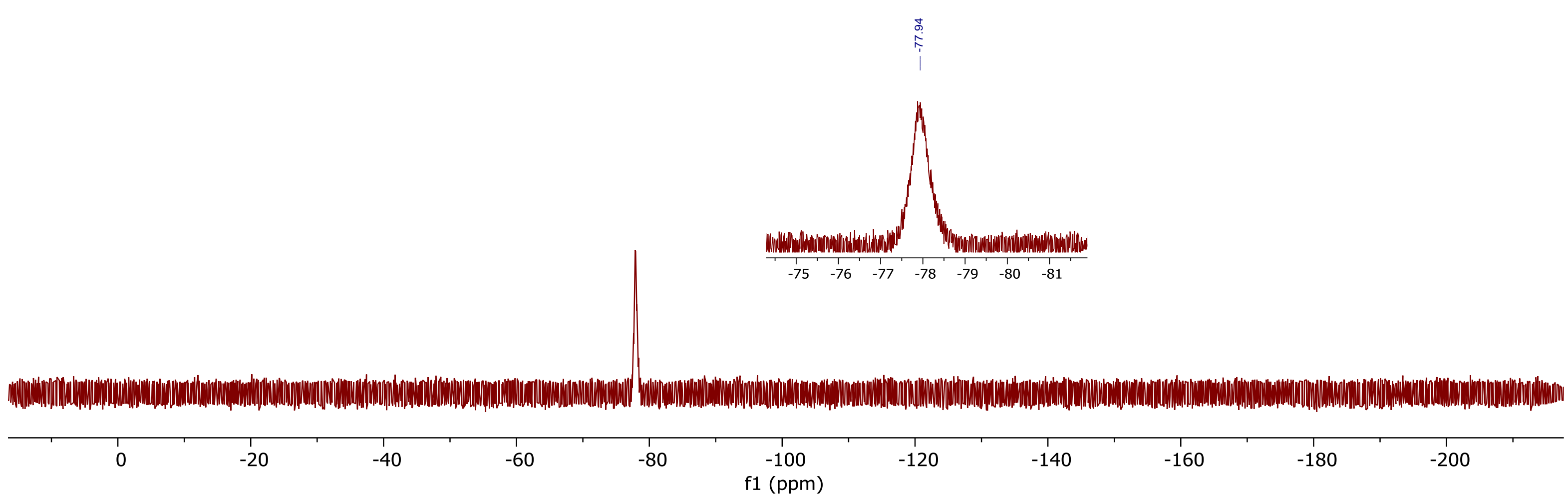

Figure S7. ^{1}H NMR spectrum of $[(dcpe)Ni(\mu\text{-}F)]_2[BF_4]_2$ (**5**) in CD_2Cl_2.

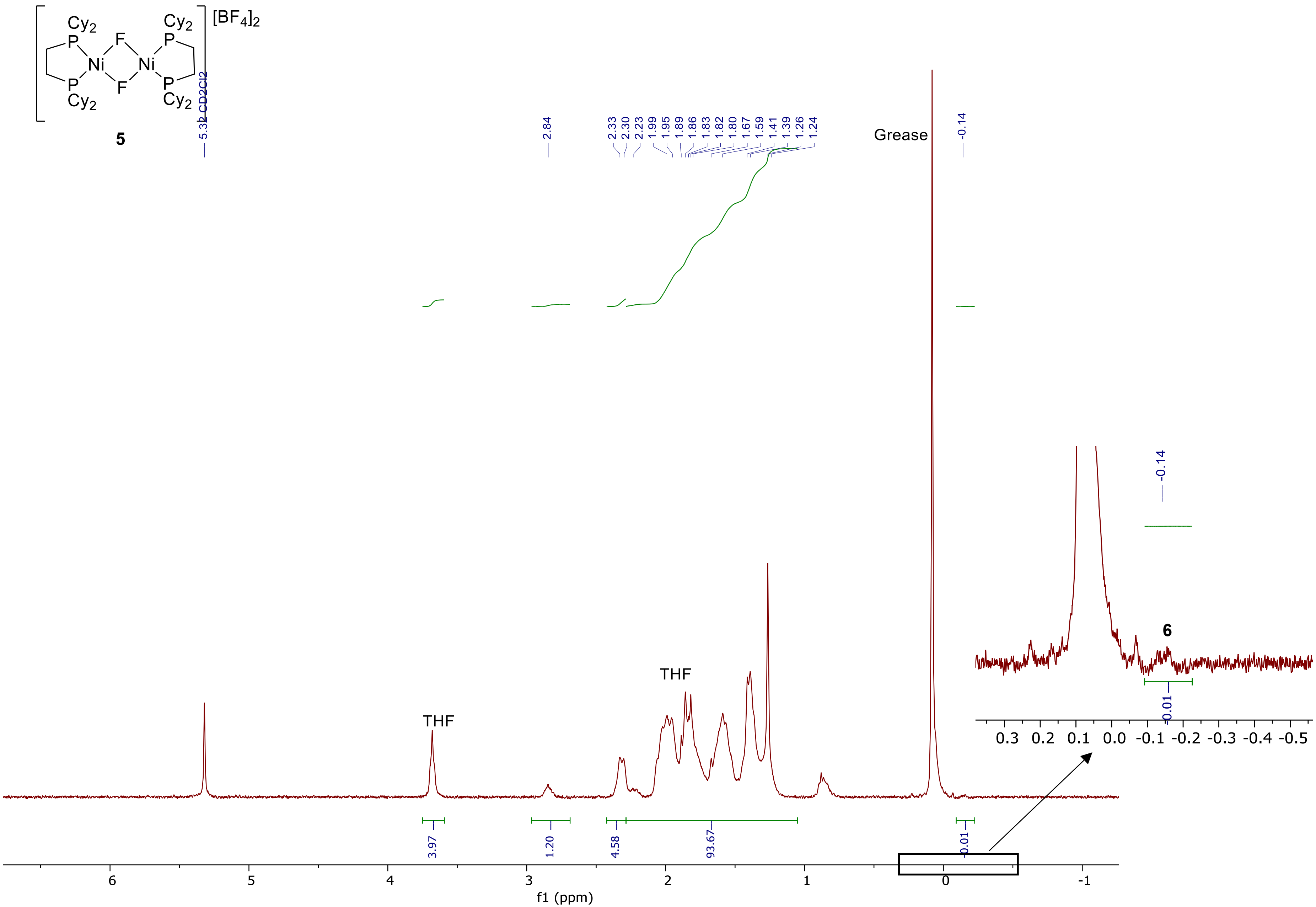

Figure S8. $^{31}P\{^{1}H\}$ NMR spectrum of a mixture of $[(dcpe)Ni(\mu\text{-}F)]_2[BF_4]_2$ (**5**) and $[(dcpe)Ni(\mu\text{-}F)(\mu\text{-}OH)Ni(dcpe)][BF_4]_2$ (**6**) in CD_2Cl_2.

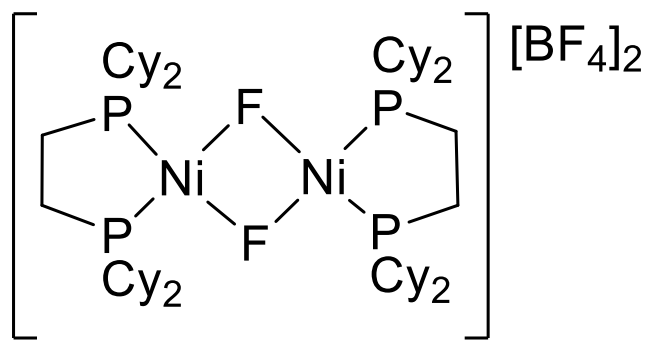

5

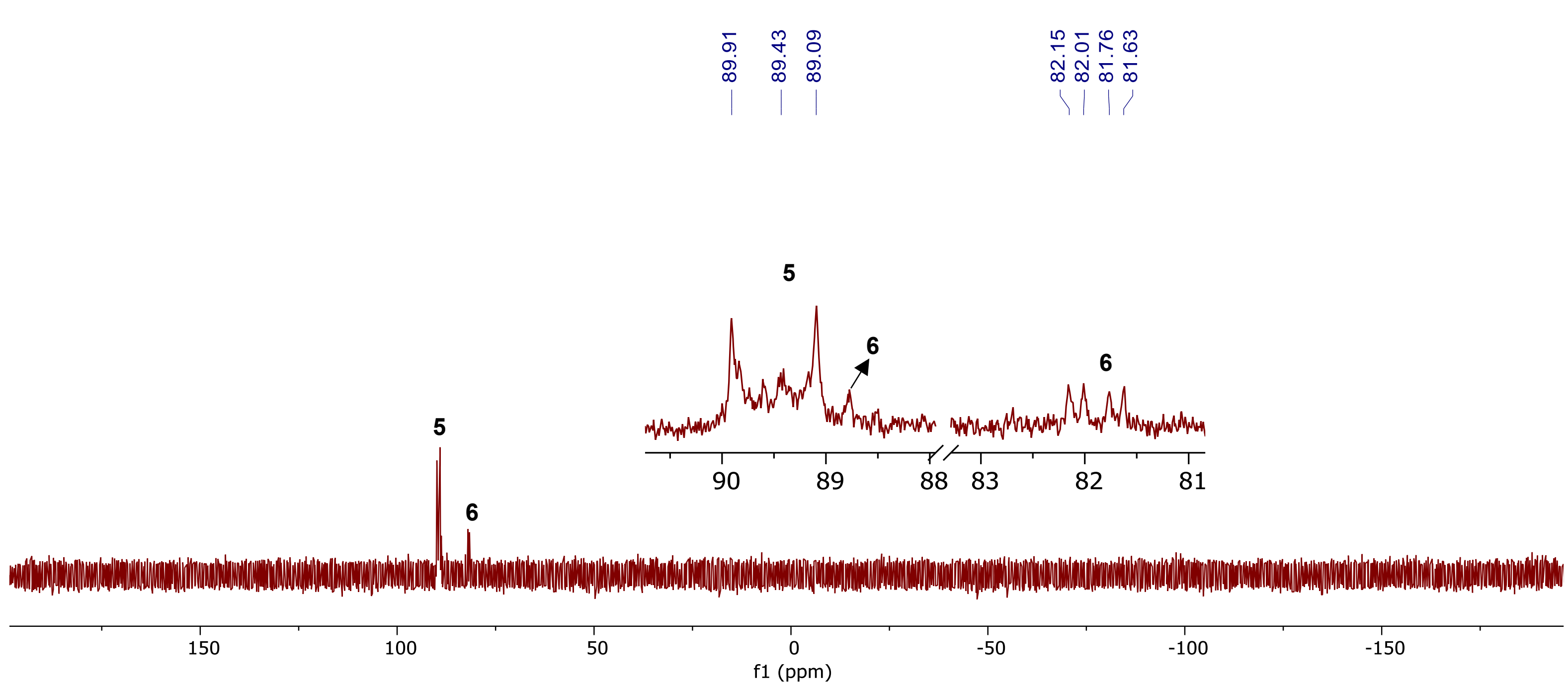

Figure S9. ^{19}F NMR spectrum of $[(dcpe)Ni(\mu\text{-}F)]_2[BF_4]_2$ (**5**) in CD_2Cl_2 (wide scan).

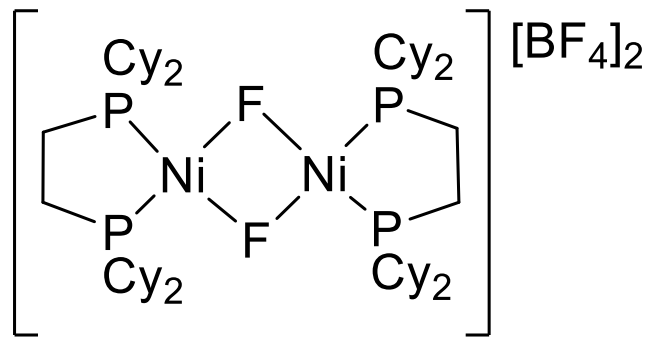

5

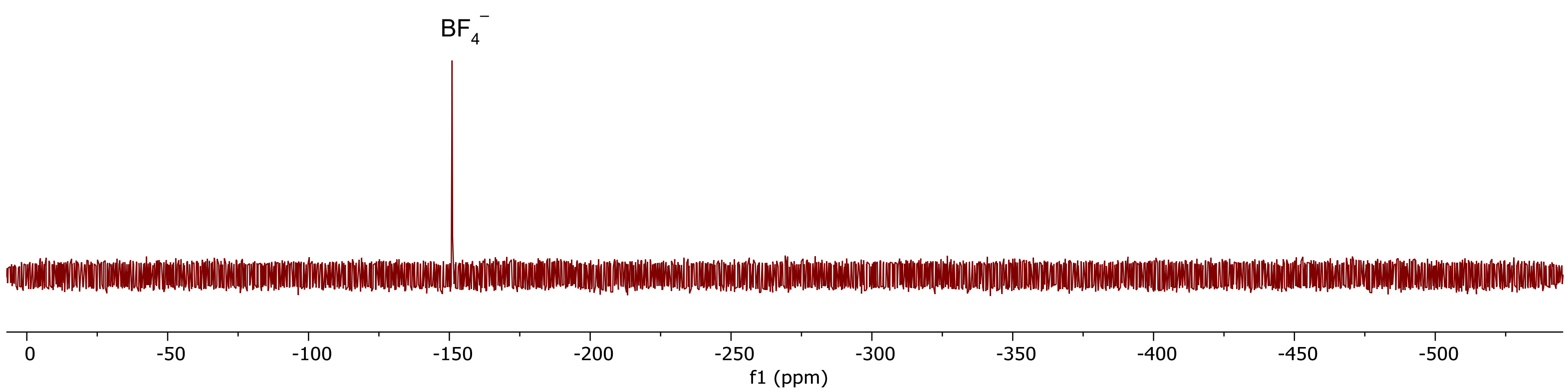

Figure S10. ^{19}F NMR (narrow scan centered at δ = –450) spectrum of [(dcpe)Ni(μ-F)]$_2$[BF$_4$]$_2$ (**5**) and [(dcpe)Ni(μ-F)(μ-OH)Ni(dcpe)][BF$_4$]$_2$ (**6**) in CD_2Cl_2.

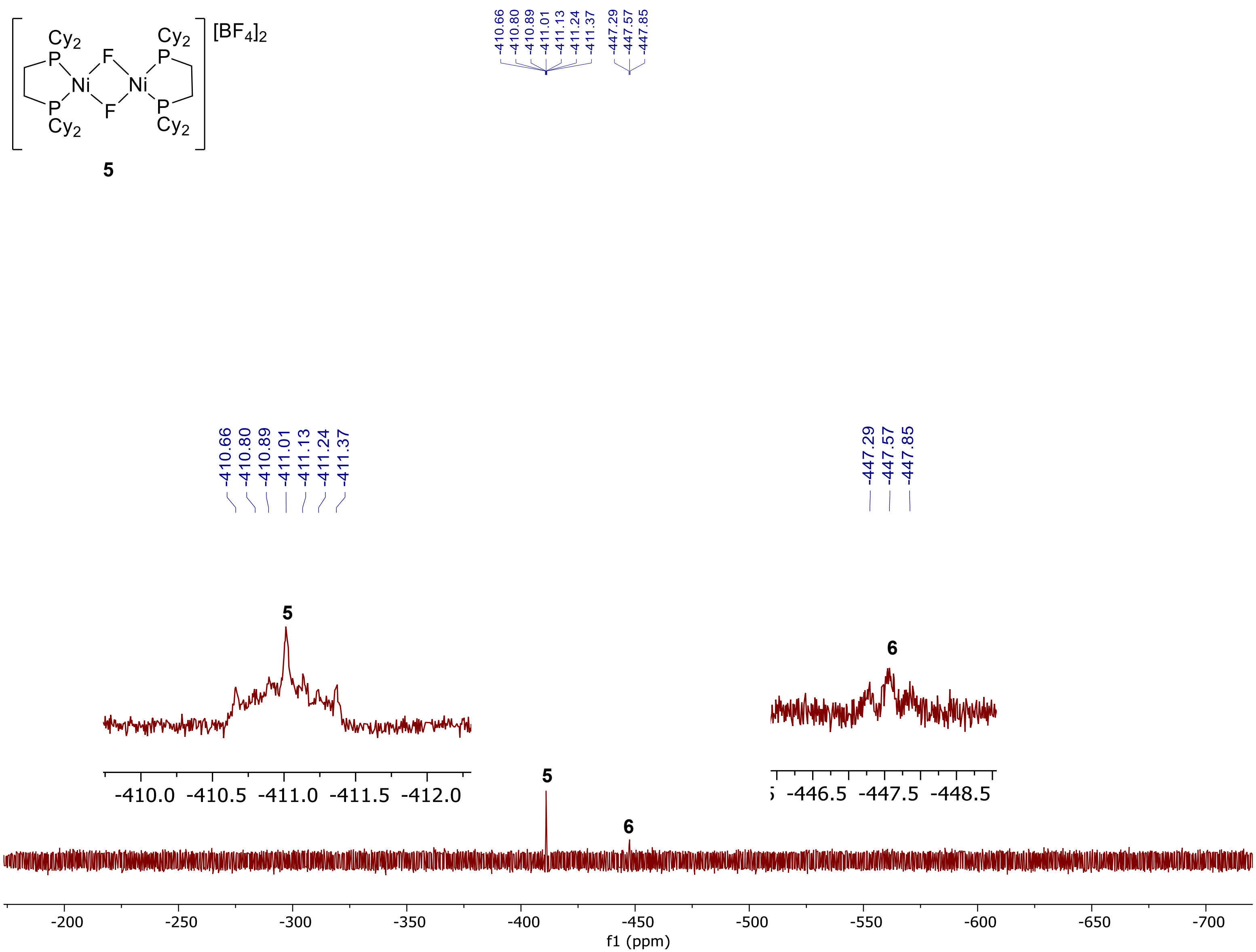

Figure S11. ^{1}H NMR spectrum of $[Ni(Cl)_2(dcpe)]$ (**8**) in CD_2Cl_2.

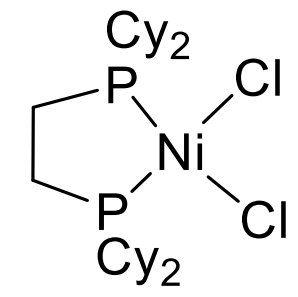

8

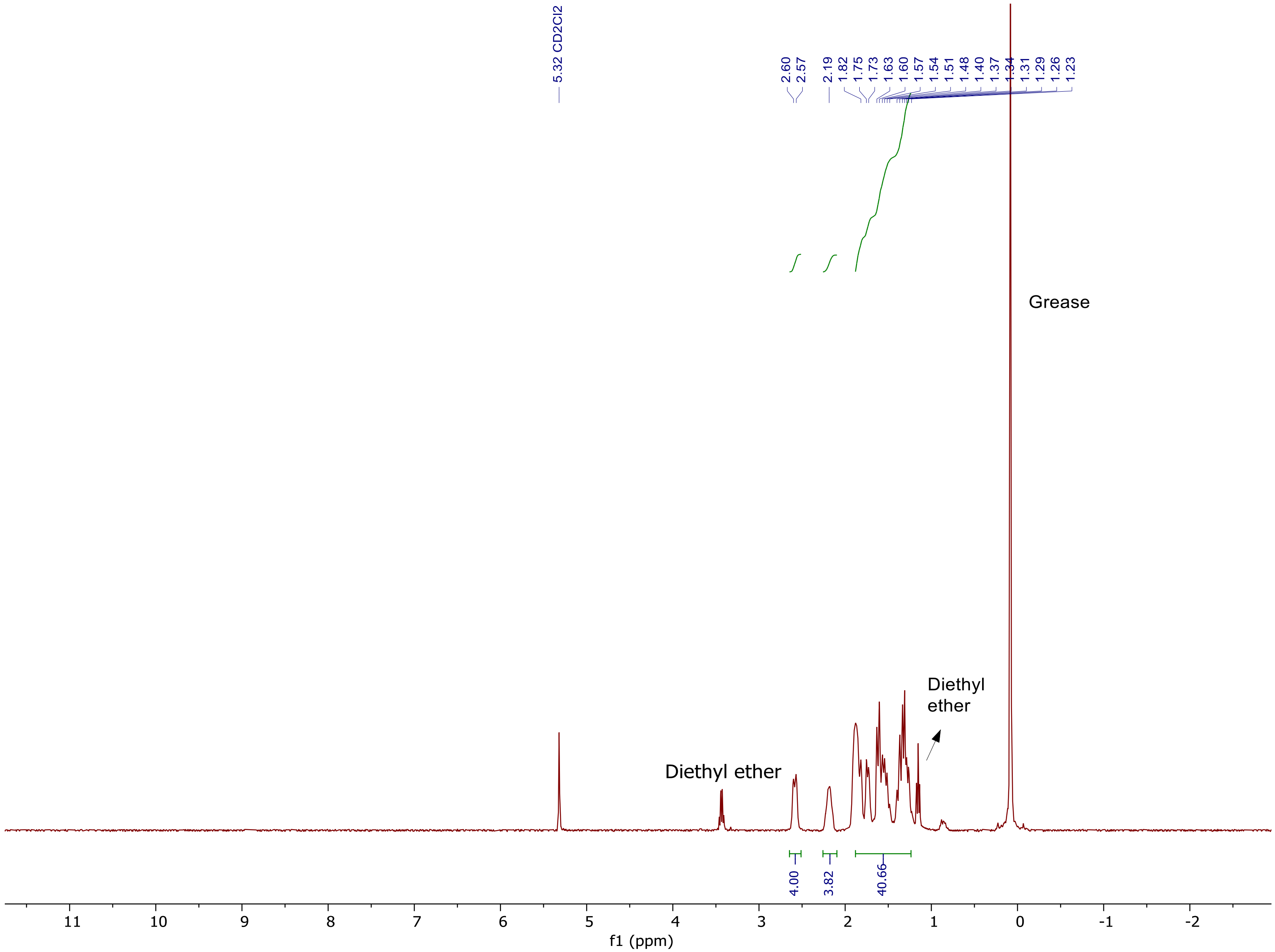

Figure S12. $^{31}P\{^{1}H\}$ NMR spectrum of $[Ni(Cl)_2(dcpe)]$ (**8**) in CD_2Cl_2.

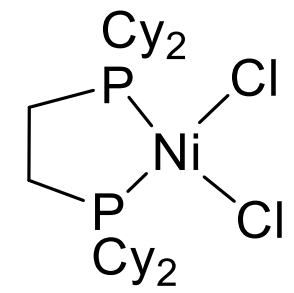

8

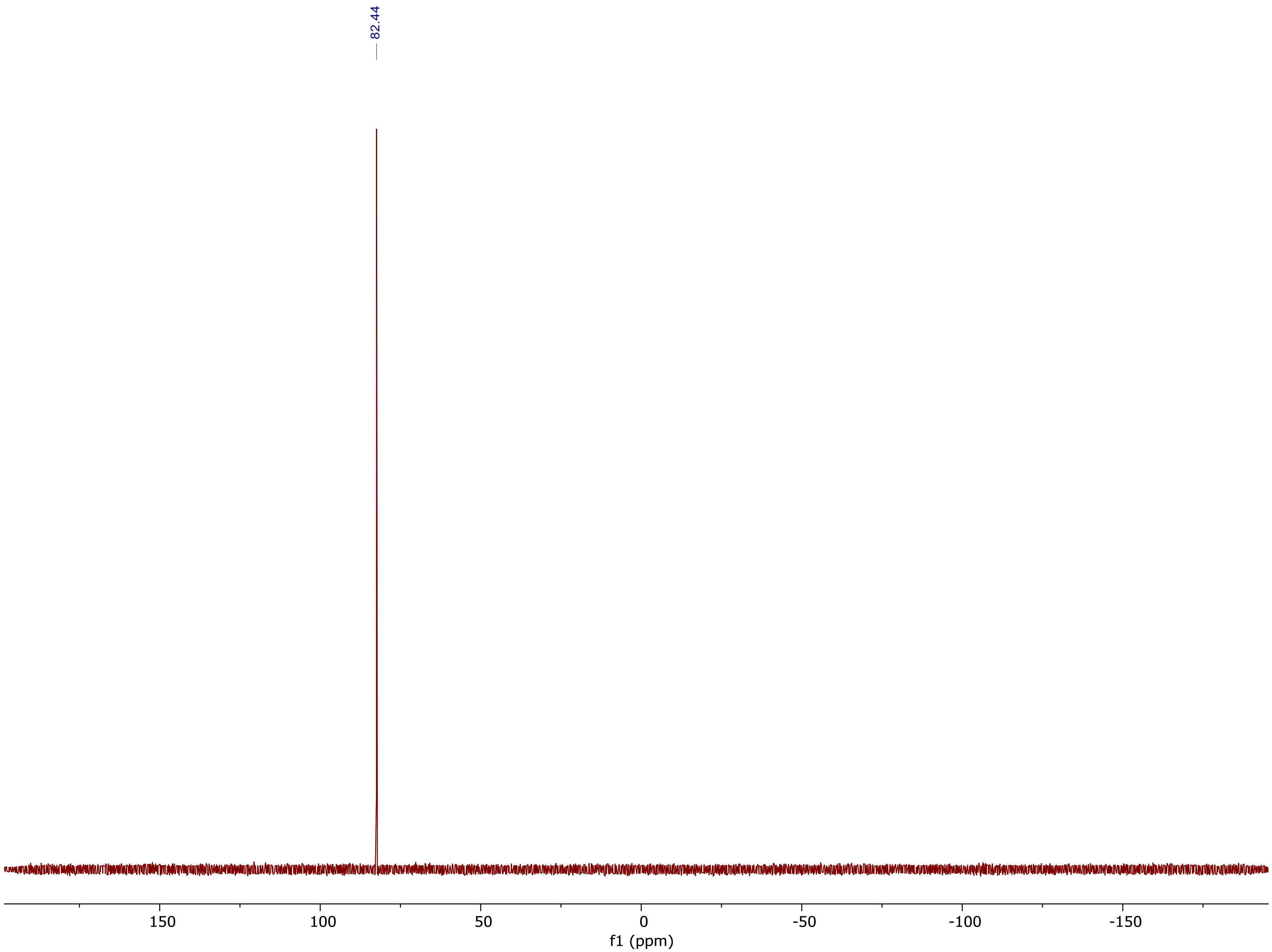

Figure S13. ^{1}H NMR spectrum of the decomposition of [(dcpe)Ni(*μ*-F)]$_2$[BF$_4$]$_2$ (**5**) to [(dcpe)$_2$Ni$_2$(*μ*-F) (*μ*-OH)][BF$_4$]$_2$ (**6**) and [(dcpe)Ni(*μ*-OH)]$_2$[BF$_4$]$_2$ (**7a**) in the presence of trace amount of water in CD$_2$Cl$_2$.

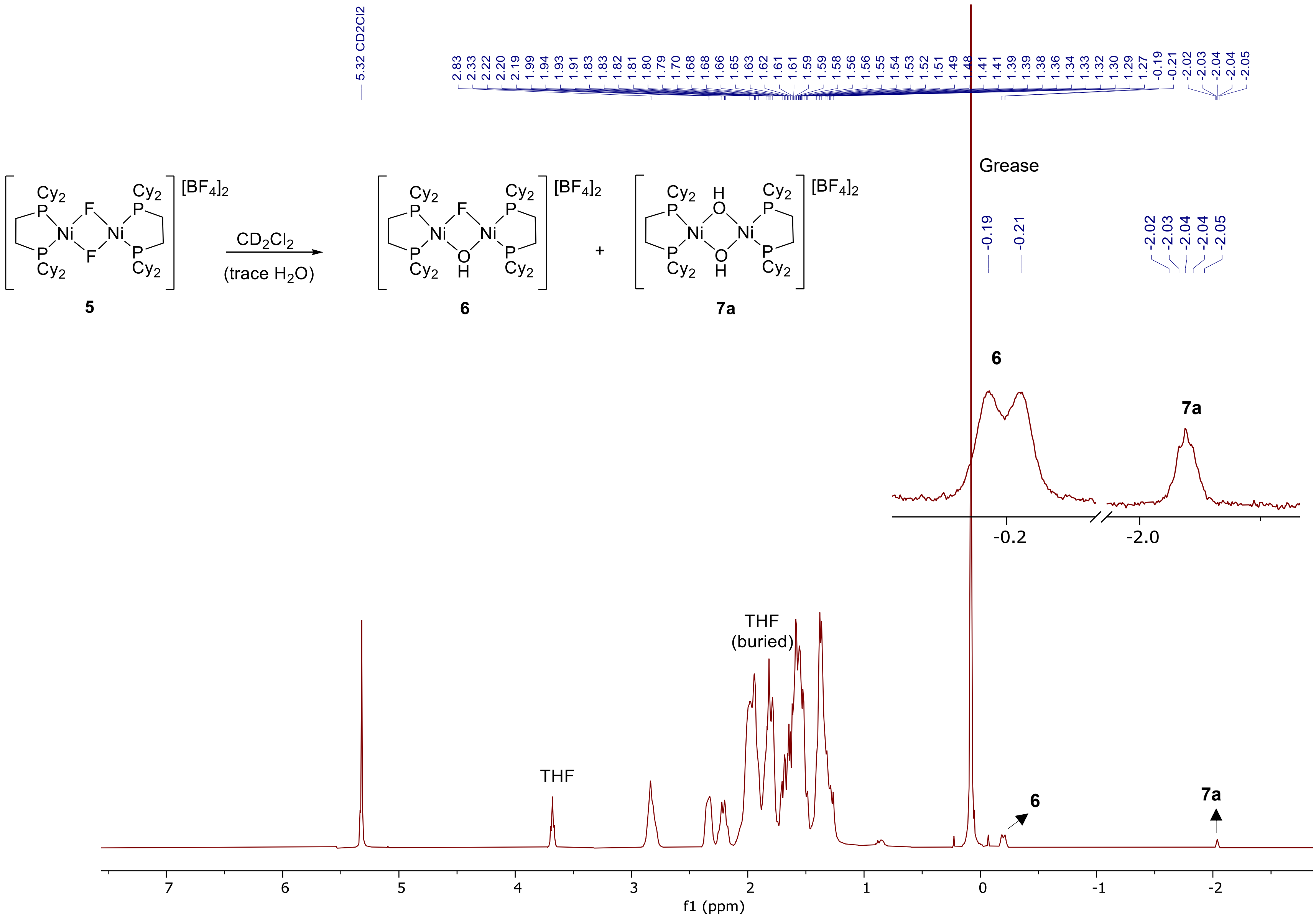

Figure S14. $^{1}H\{^{19}F\}$ NMR spectrum of the decomposition of [(dcpe)Ni(μ-F)]$_2$[BF$_4$]$_2$ (**5**) to [(dcpe)$_2$Ni$_2$(μ-F) (μ-OH)][BF$_4$]$_2$ (**6**) and [(dcpe)Ni(μ-OH)]$_2$[BF$_4$]$_2$ (**7a**) in the presence of trace amount of water in CD_2Cl_2.

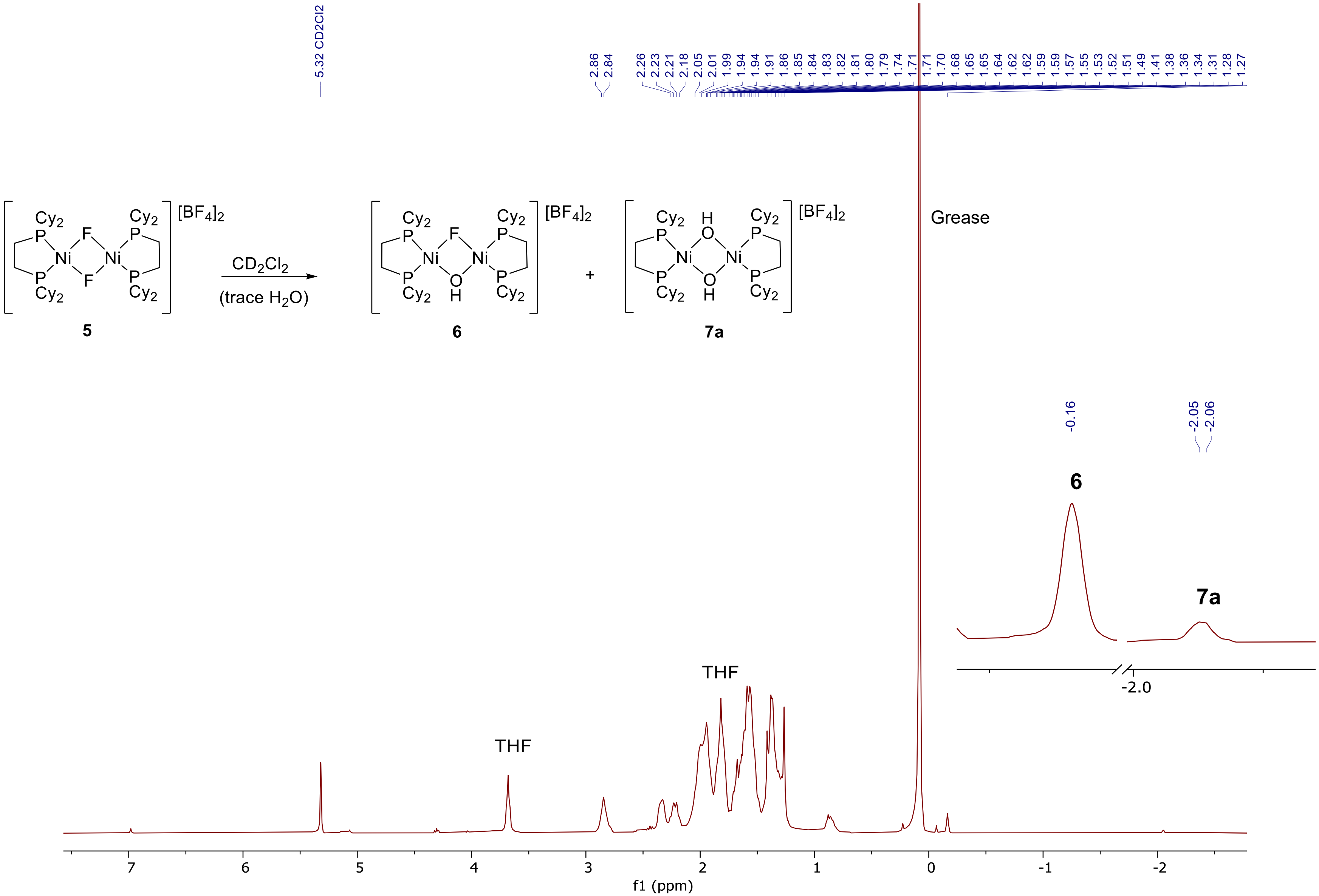

Figure S15. ¹H{³¹P} NMR spectrum of the decomposition of [(dcpe)Ni(μ-F)]$_2$[BF$_4$]$_2$ (**5**) to [(dcpe)$_2$Ni$_2$(μ-F) (μ-OH)][BF$_4$]$_2$ (**6**) and [(dcpe)Ni(μ-OH)]$_2$[BF$_4$]$_2$ (**7a**) in the presence of trace amount of water in CD_2Cl_2.

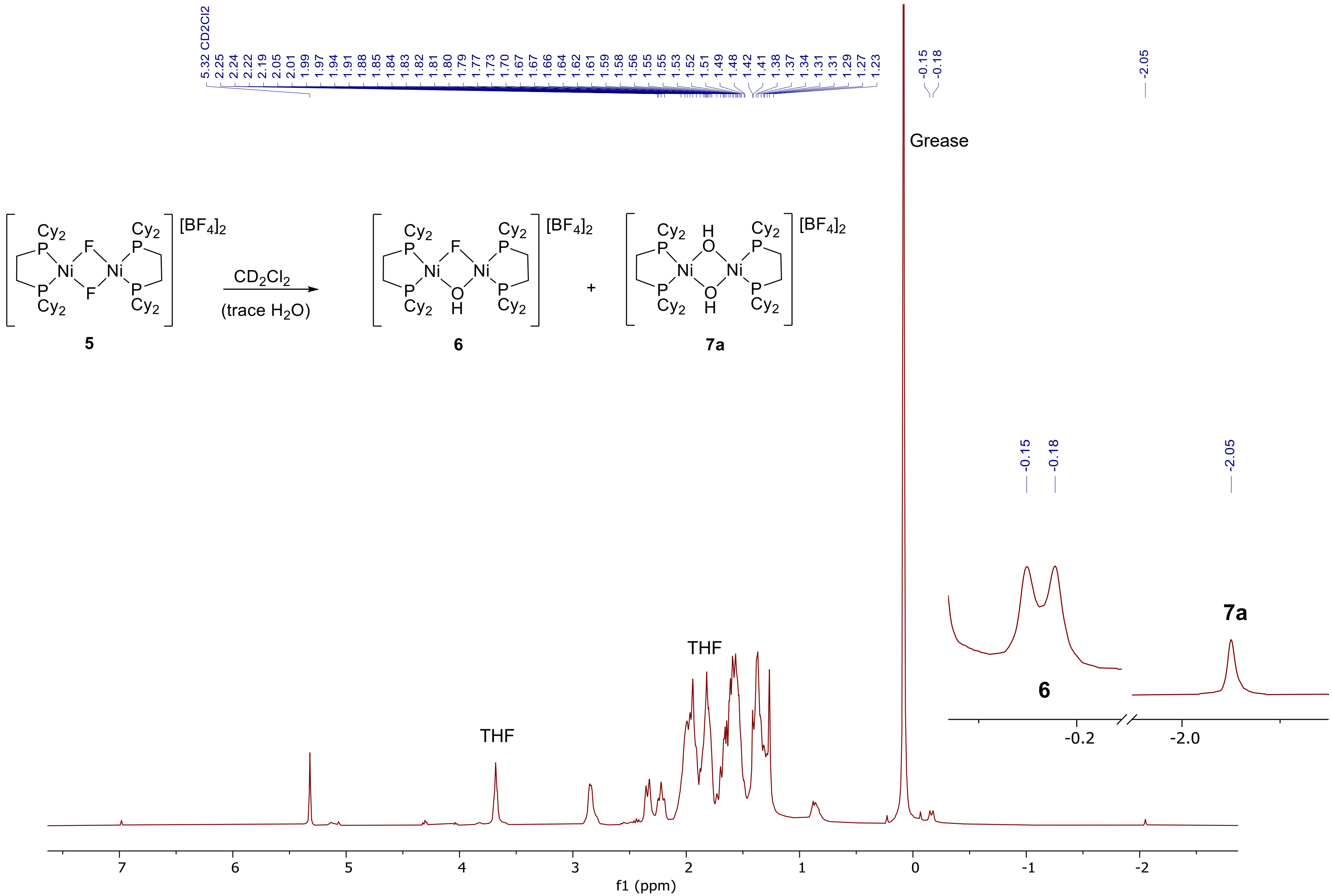

Figure S16. $^{31}P\{^{1}H\}$ NMR spectrum of the decomposition of $[(dcpe)Ni(\mu\text{-}F)]_2[BF_4]_2$ (**5**) to $[(dcpe)_2Ni_2(\mu\text{-}F)(\mu\text{-}OH)][BF_4]_2$ (**6**) and $[(dcpe)Ni(\mu\text{-}OH)]_2[BF_4]_2$ (**7a**) in the presence of trace amount of water in CD_2Cl_2.

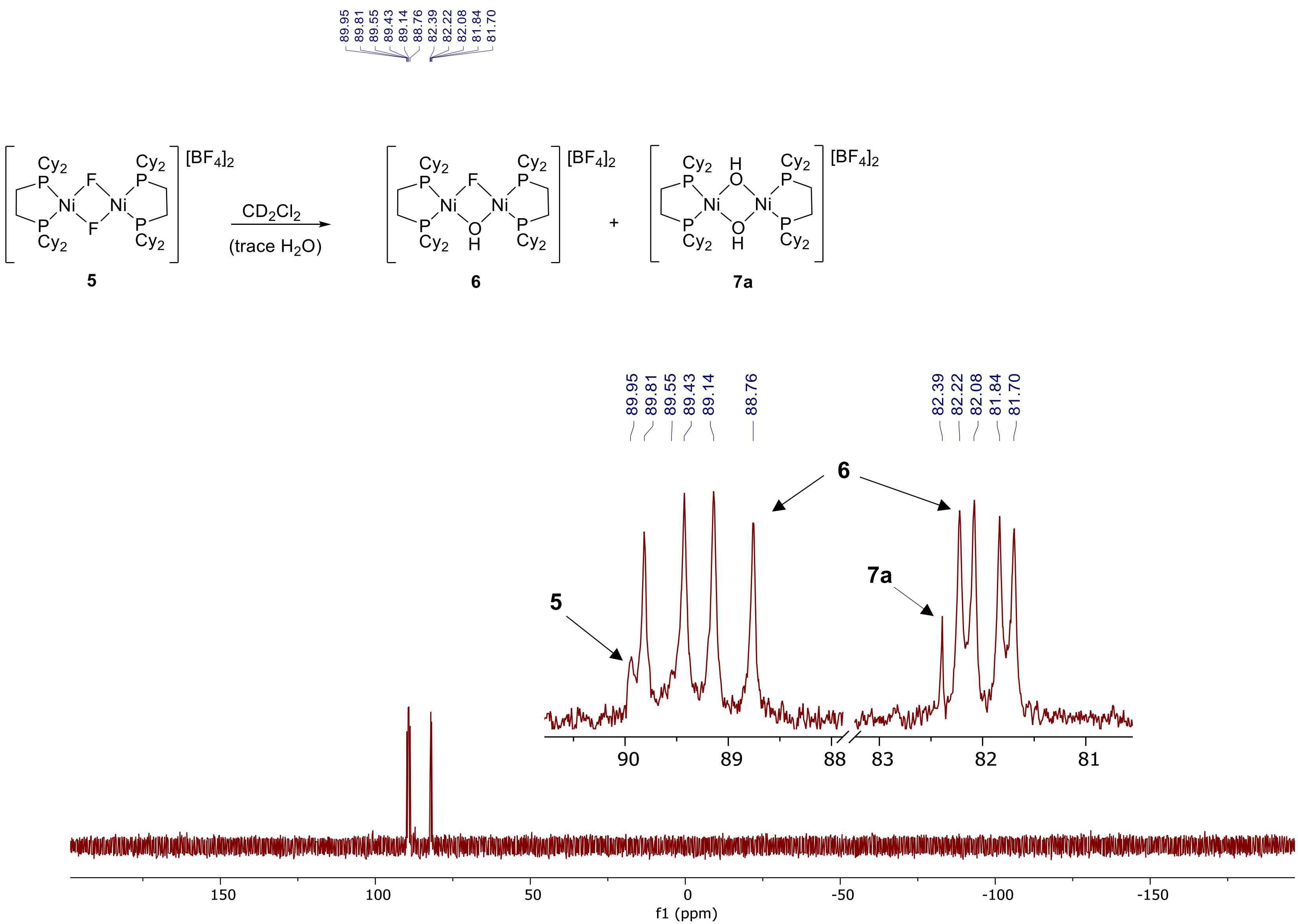

Figure S17. ^{19}F NMR spectrum (wide scan) of the decomposition of $[(dcpe)Ni(\mu\text{-}F)]_2[BF_4]_2$ (**5**) to $[(dcpe)_2Ni_2(\mu\text{-}F)(\mu\text{-}OH)][BF_4]_2$ (**6**) and $[(dcpe)Ni(\mu\text{-}OH)]_2[BF_4]_2$ (**7a**) in the presence of trace amount of water in CD_2Cl_2.

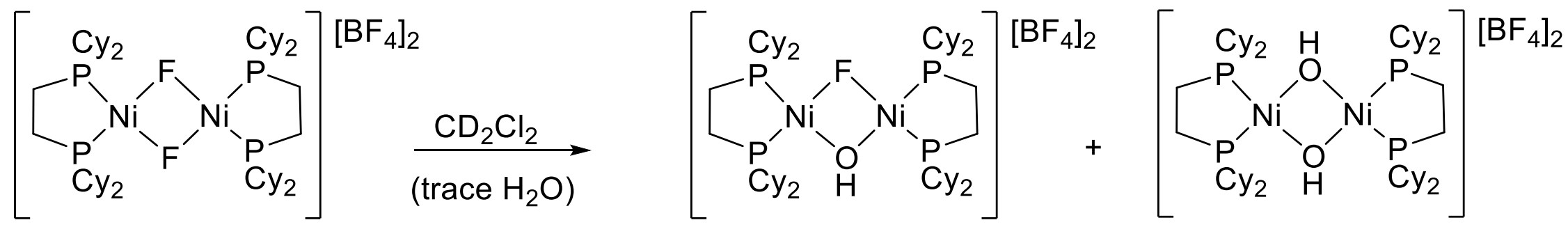

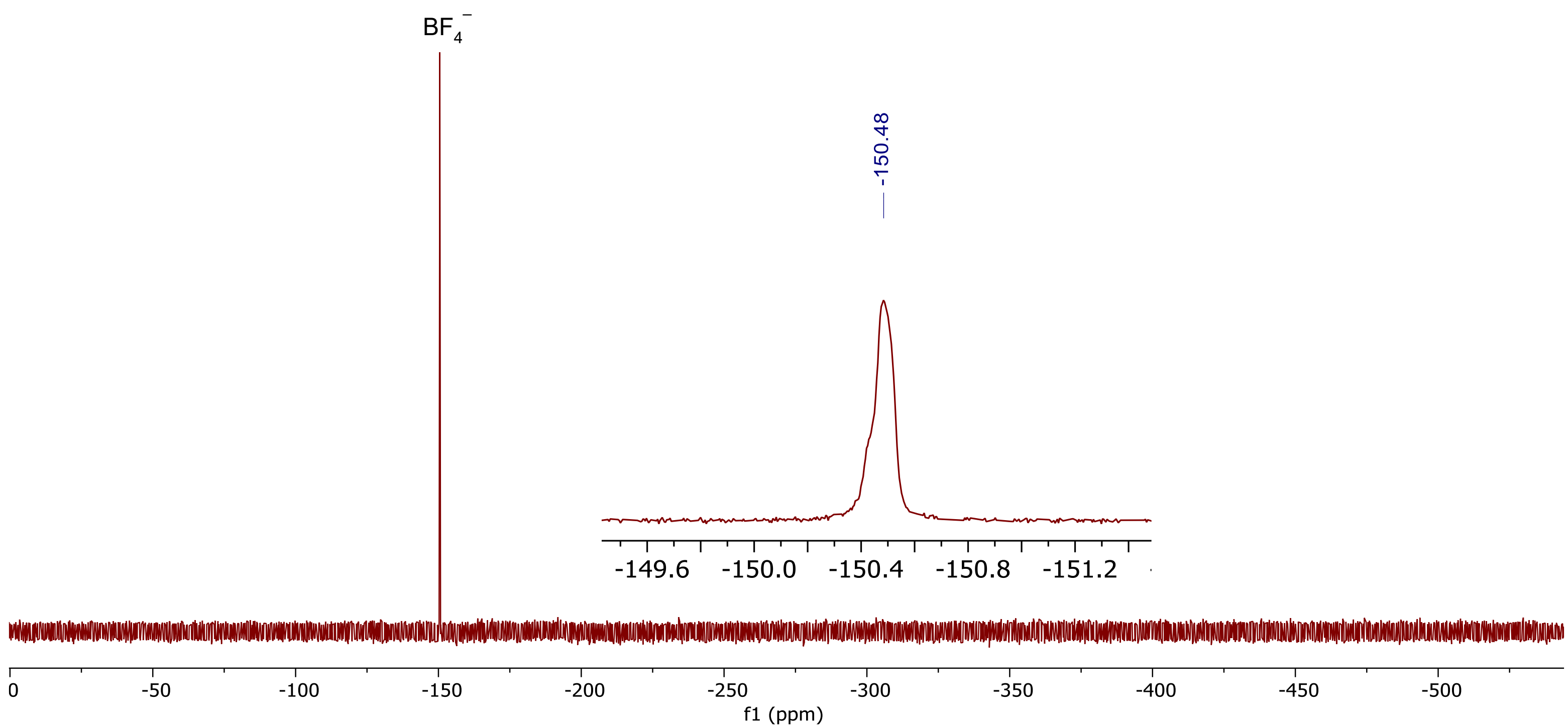

Figure S18. ^{19}F NMR spectrum(narrow scan centered at δ = –450) of the decomposition of [(dcpe)Ni(μ-F)]$_2$[BF$_4$]$_2$ (**5**) to [(dcpe)$_2$Ni$_2$(μ-F) (μ-OH)][BF$_4$]$_2$ (**6**) and [(dcpe)Ni(μ-OH)]$_2$[BF$_4$]$_2$ (**7a**) in the presence of trace amount of water in CD_2Cl_2.

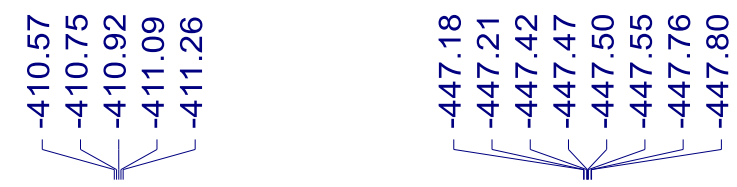

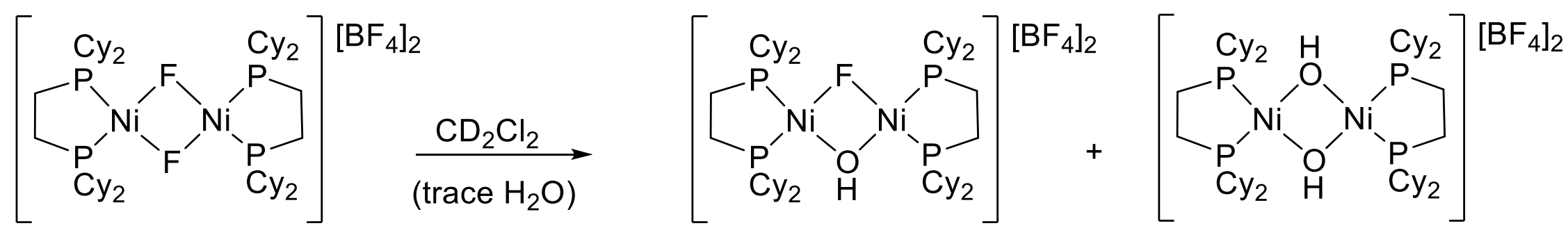

5 **6** **7a**

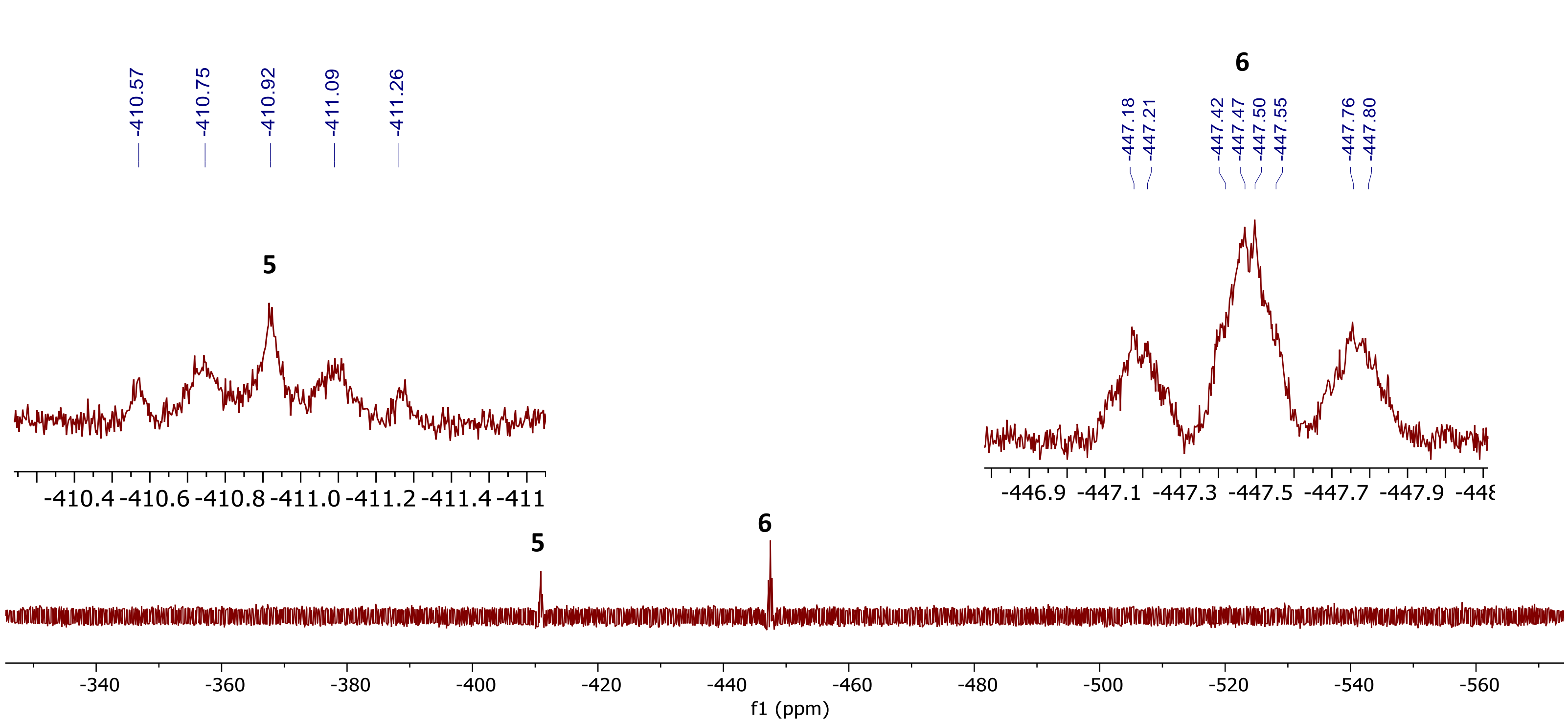

Figure S19. $^{19}F\{^1H\}$ NMR spectrum of the decomposition of [(dcpe)Ni(μ-F)]$_2$[BF$_4$]$_2$ (**5**) to [(dcpe)$_2$Ni$_2$(μ-F) (μ-OH)][BF$_4$]$_2$ (**6**) and [(dcpe)Ni(μ-OH)]$_2$[BF$_4$]$_2$ (**7a**) in the presence of trace amount of water in CD_2Cl_2.

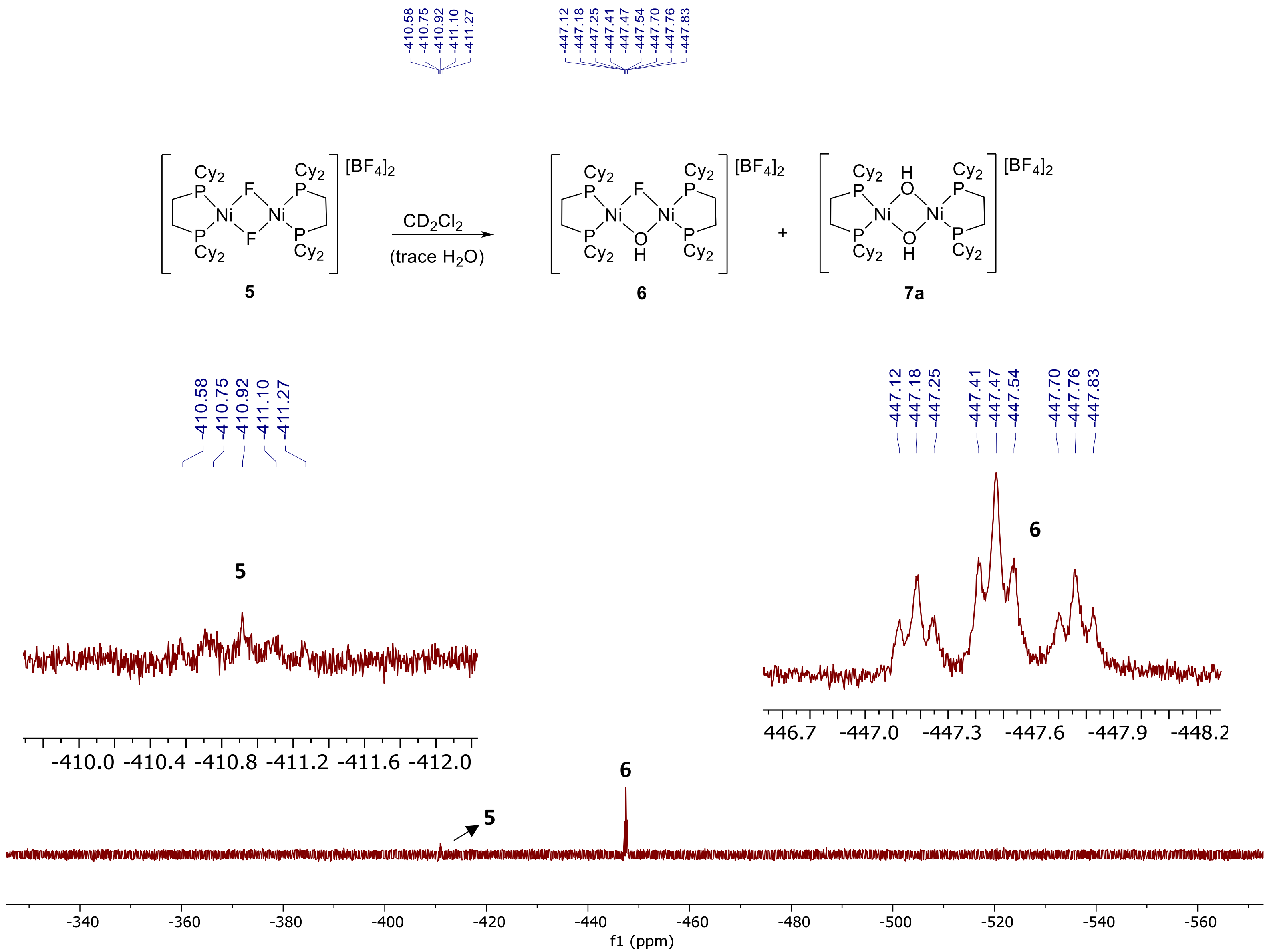

Figure S20 $^{31}P\{^1H\}$ - ^1H HMBC of the decomposition of [(dcpe)Ni(μ-F)]$_2$[BF$_4$]$_2$ (**5**) to [(dcpe)$_2$Ni$_2$(μ-F) (μ-OH)][BF$_4$]$_2$ (**6**) and [(dcpe)Ni(μ-OH)]$_2$[BF$_4$]$_2$ (**7a**) in the presence of trace amount of water in CD_2Cl_2.

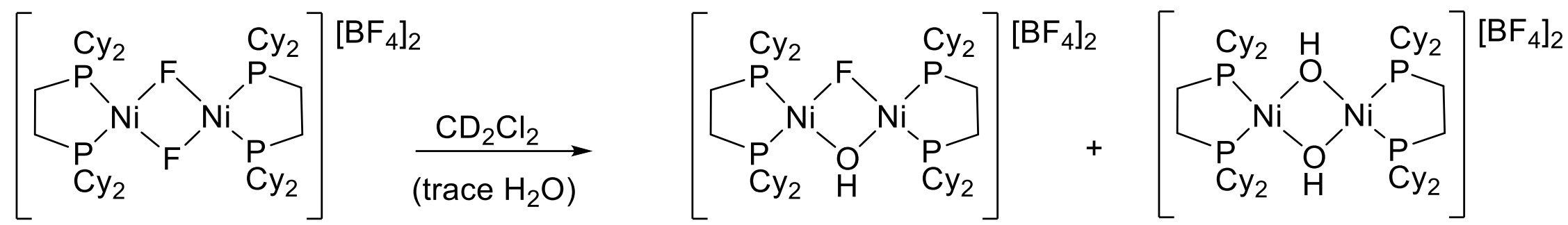

5 **6** **7a**

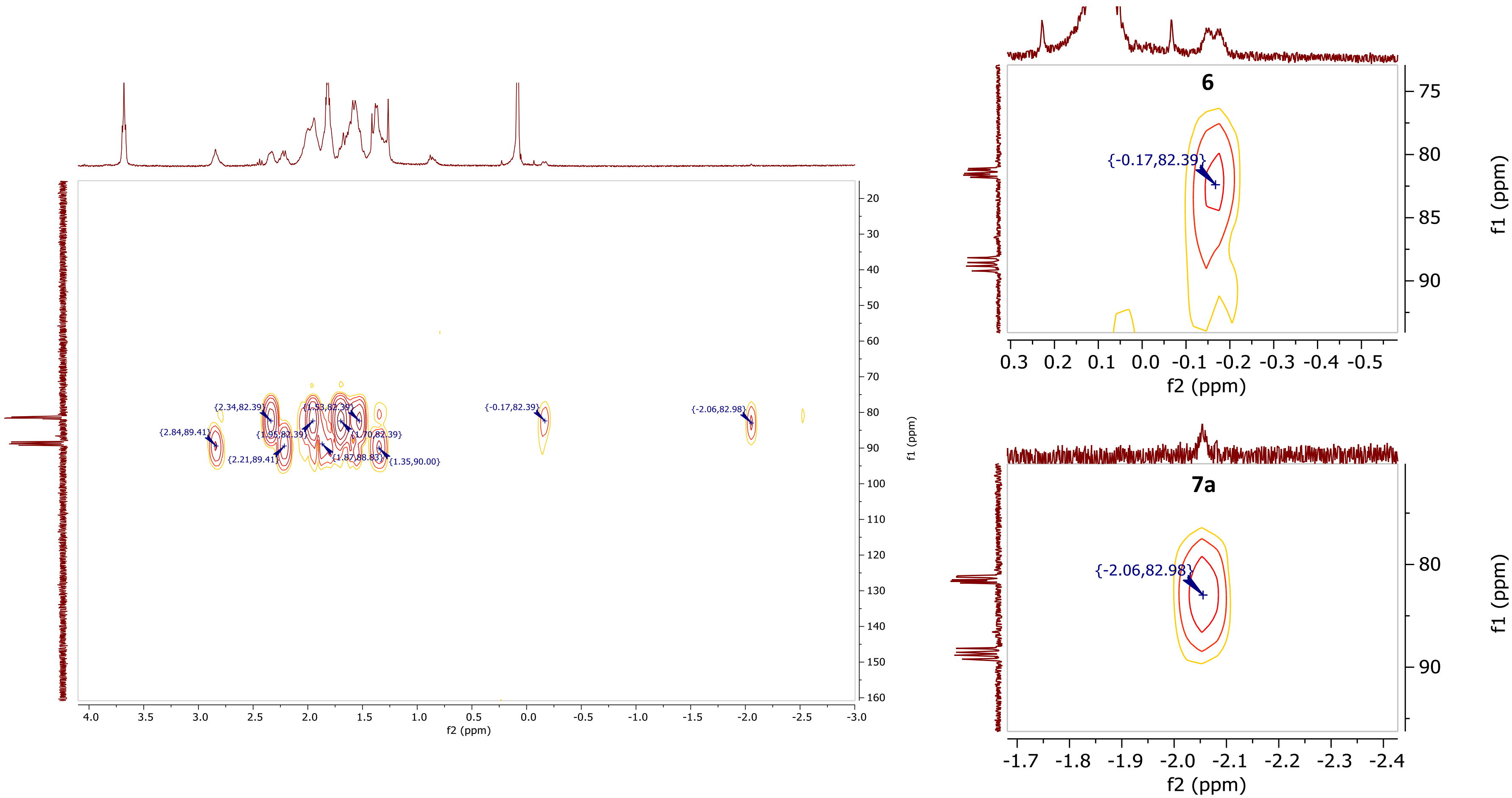

Figure S21 ^1H NMR spectrum of [(dcpe)Ni(μ-OH)]$_2$[OTf]$_2$ (**7b**) and its decomposition product [(dcpe)NiCl$_2$] **(8)** in CD_2Cl_2.

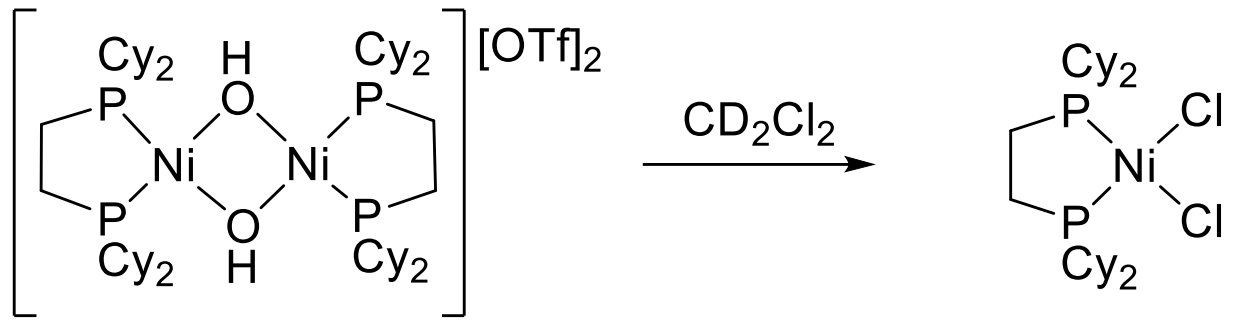

7b **8**

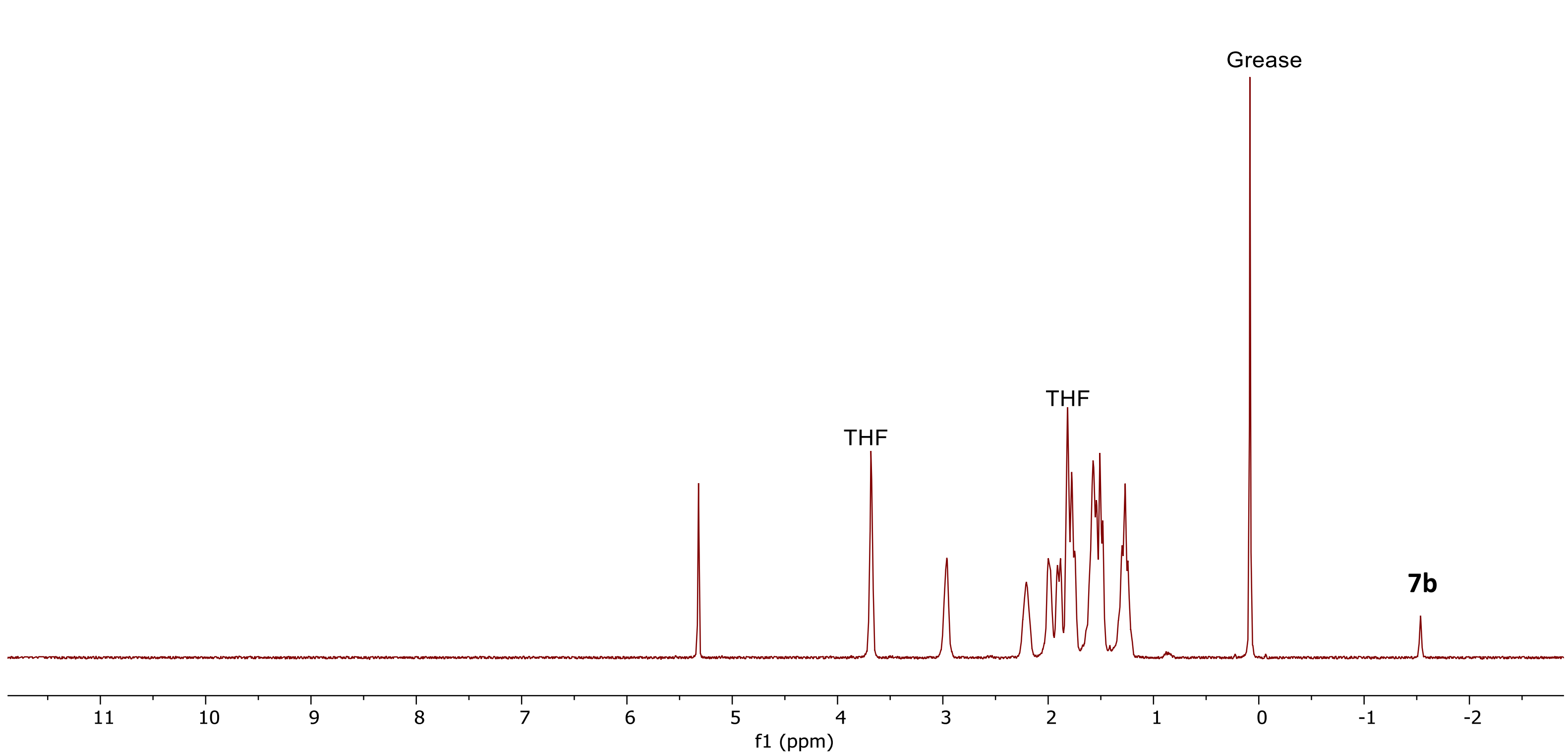

Figure S22 $^{31}P\{^1H\}$ NMR spectrum of [(dcpe)Ni(μ-OH)]$_2$[OTf]$_2$ (7**b**) and its decomposition product [(dcpe)NiCl$_2$] **(8)** in CD_2Cl_2.

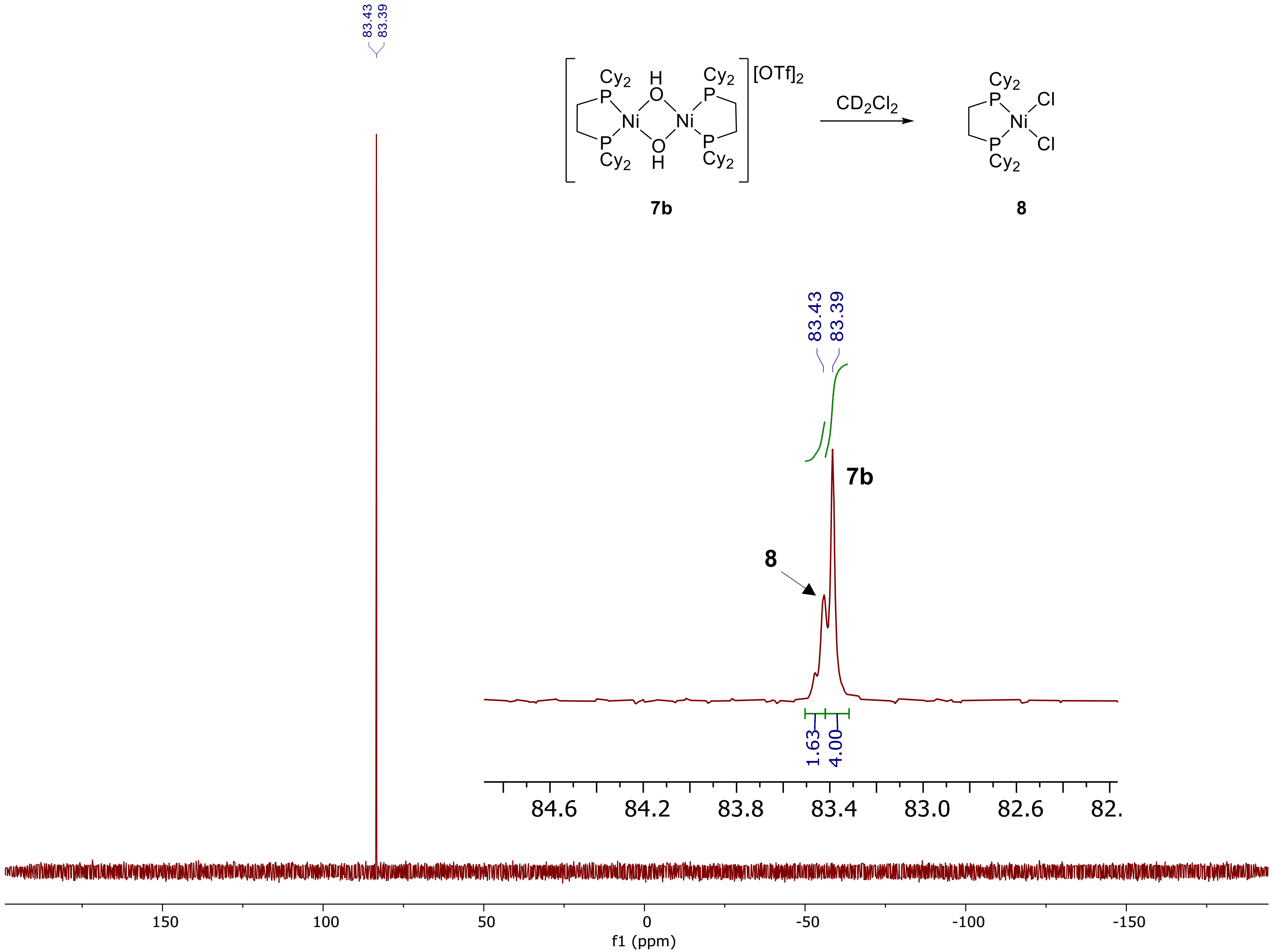

Figure S23 ^{19}F NMR NMR spectrum of [(dcpe)Ni(μ-OH)]$_2$[OTf]$_2$ (**7b**) and its decomposition product [(dcpe)NiCl$_2$] **(8)** in CD_2Cl_2.

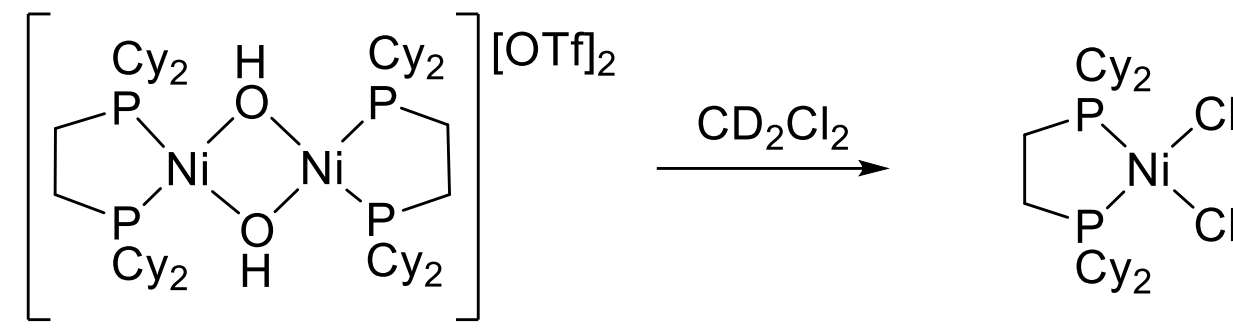

7b **8**

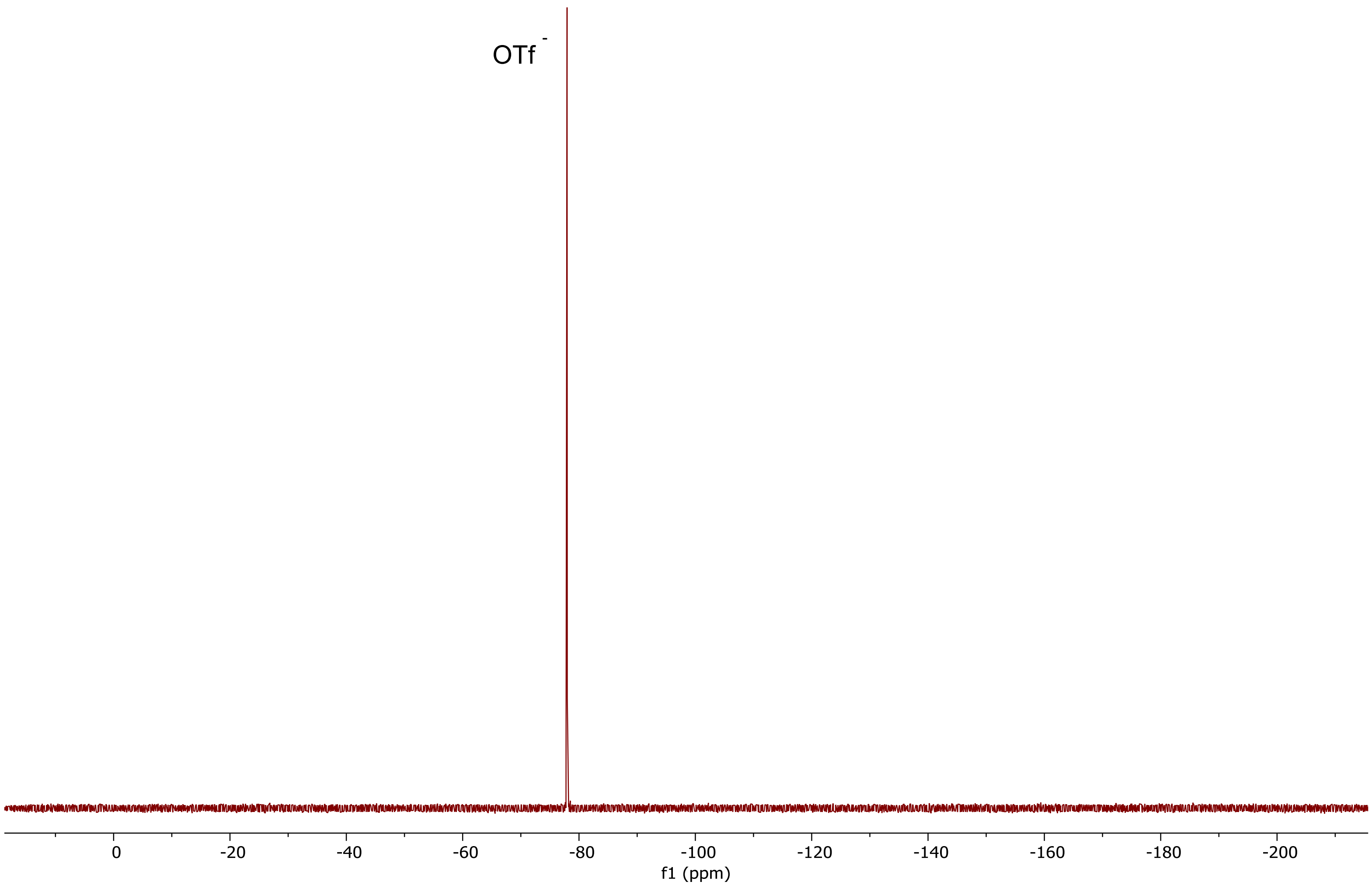

Figure S24 $^{31}P\{^{1}H\}$ – ^{1}H HMBC of [(dcpe)Ni(μ-OH)]$_2$[OTf]$_2$ (**7b**) and its decomposition product [(dcpe)$NiCl_2$] **(8)** in CD_2Cl_2.

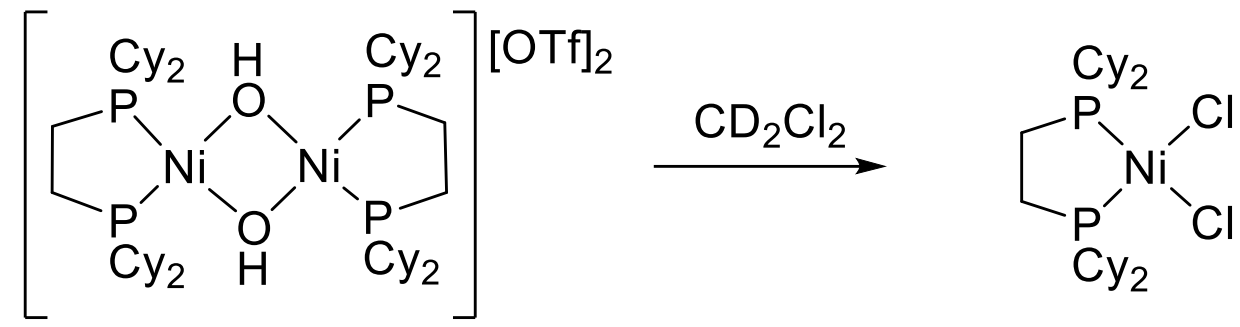

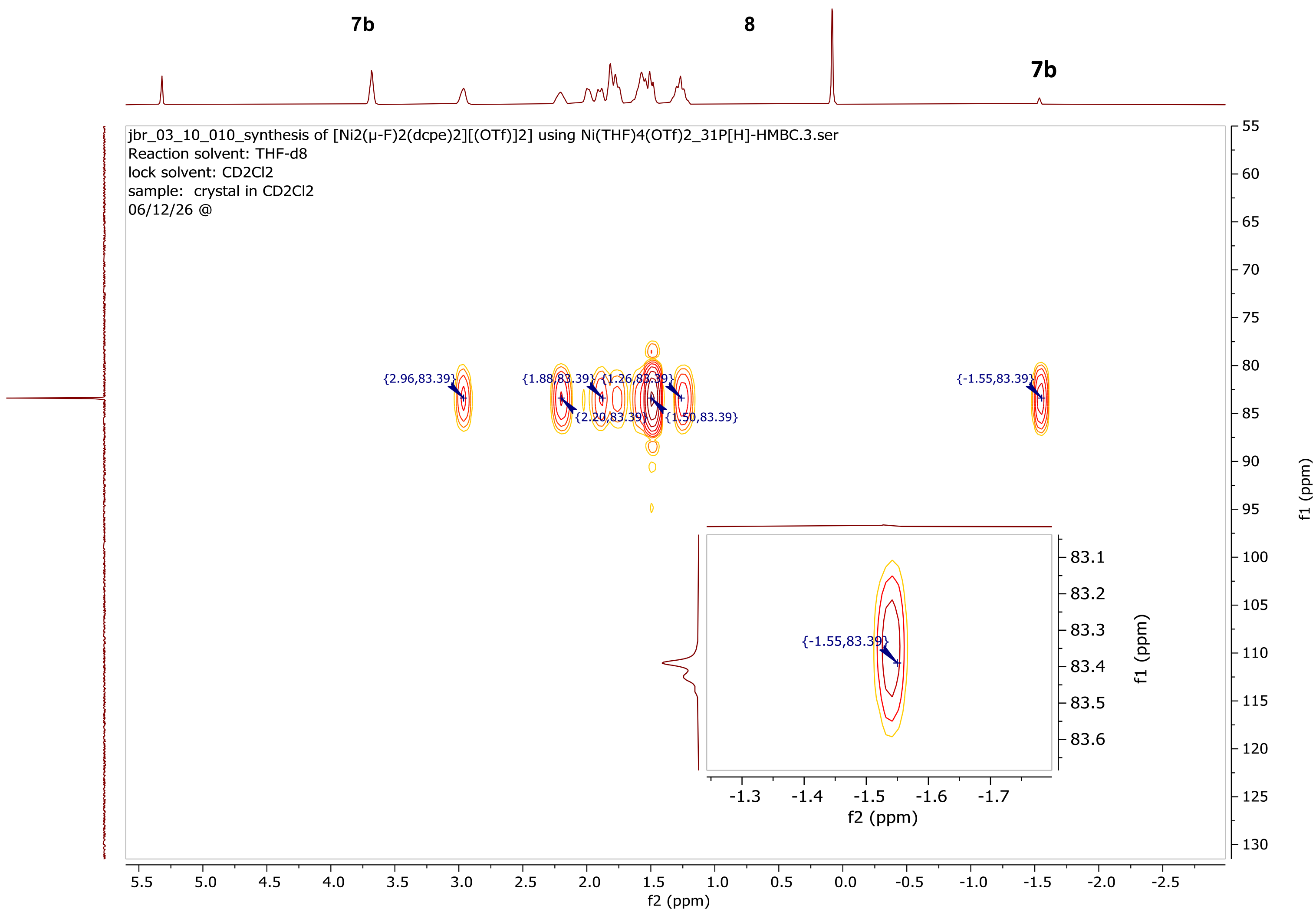

Figure S25 $^{31}P\{^{1}H\}$ monitoring of the formation of the decomposition products of [(dcpe)Ni(μ-F)]$_2$[BF$_4$]$_2$ (**5**) dissolved in CD_2Cl_2 into [(dcpe)$_2$Ni$_2$(μ-F) (μ-OH)][BF$_4$]$_2$ (**6**) and [(dcpe)Ni(μ-OH)]$_2$[BF$_4$]$_2$ (**7a**) from 0 h to 2.5 d in an air-free glass NMR tube.

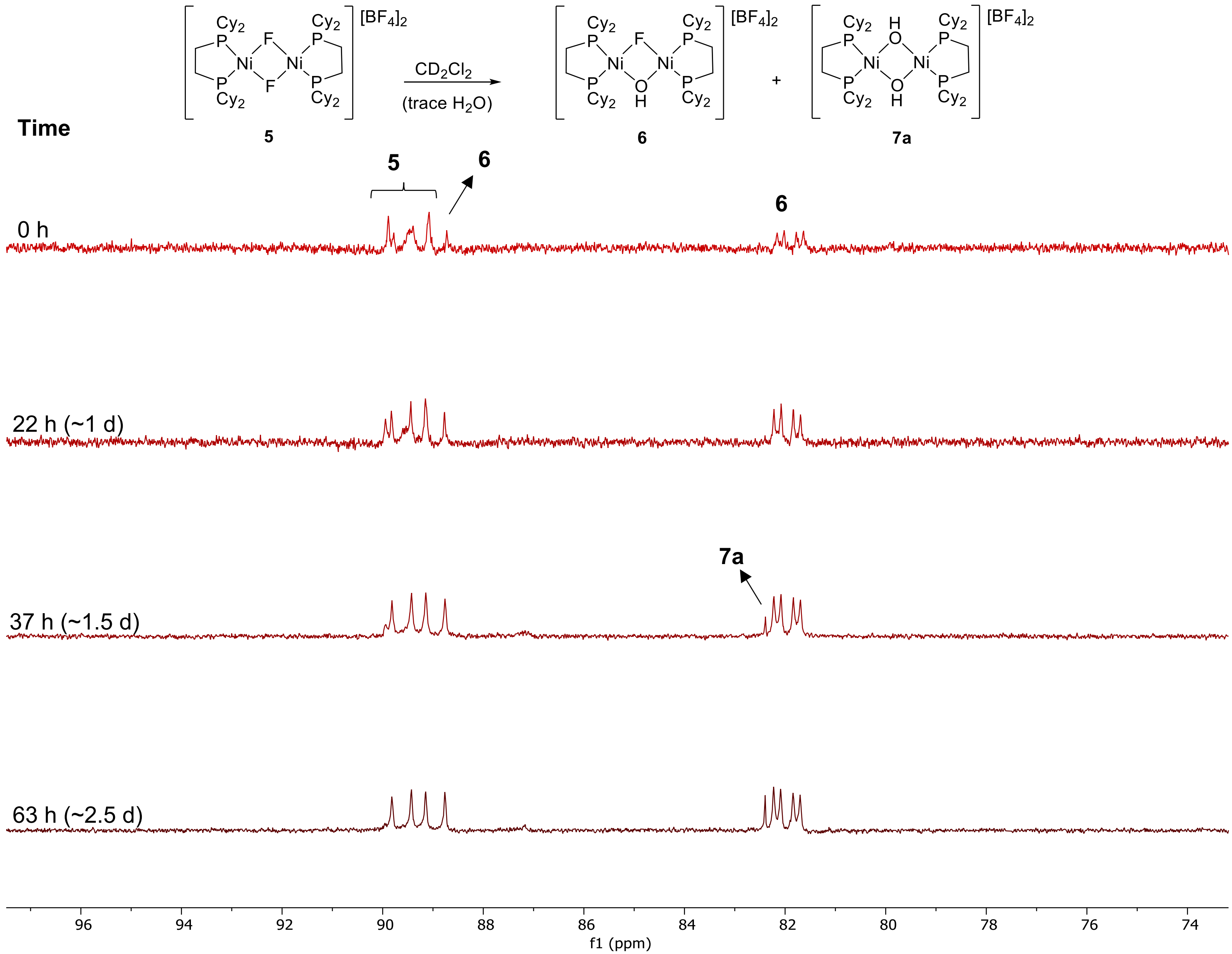

Figure S26 ^{19}F monitoring of the formation of the decomposition products of $[(dcpe)Ni(\mu\text{-}F)]_2[BF_4]_2$ (**5**) dissolved in CD_2Cl_2 into $[(dcpe)_2Ni_2(\mu\text{-}F)(\mu\text{-}OH)][BF_4]_2$ (**6**) and $[(dcpe)Ni(\mu\text{-}OH)]_2[BF_4]_2$ (**7a**) from 0 h to 2.5 d in an air-free glass NMR tube.

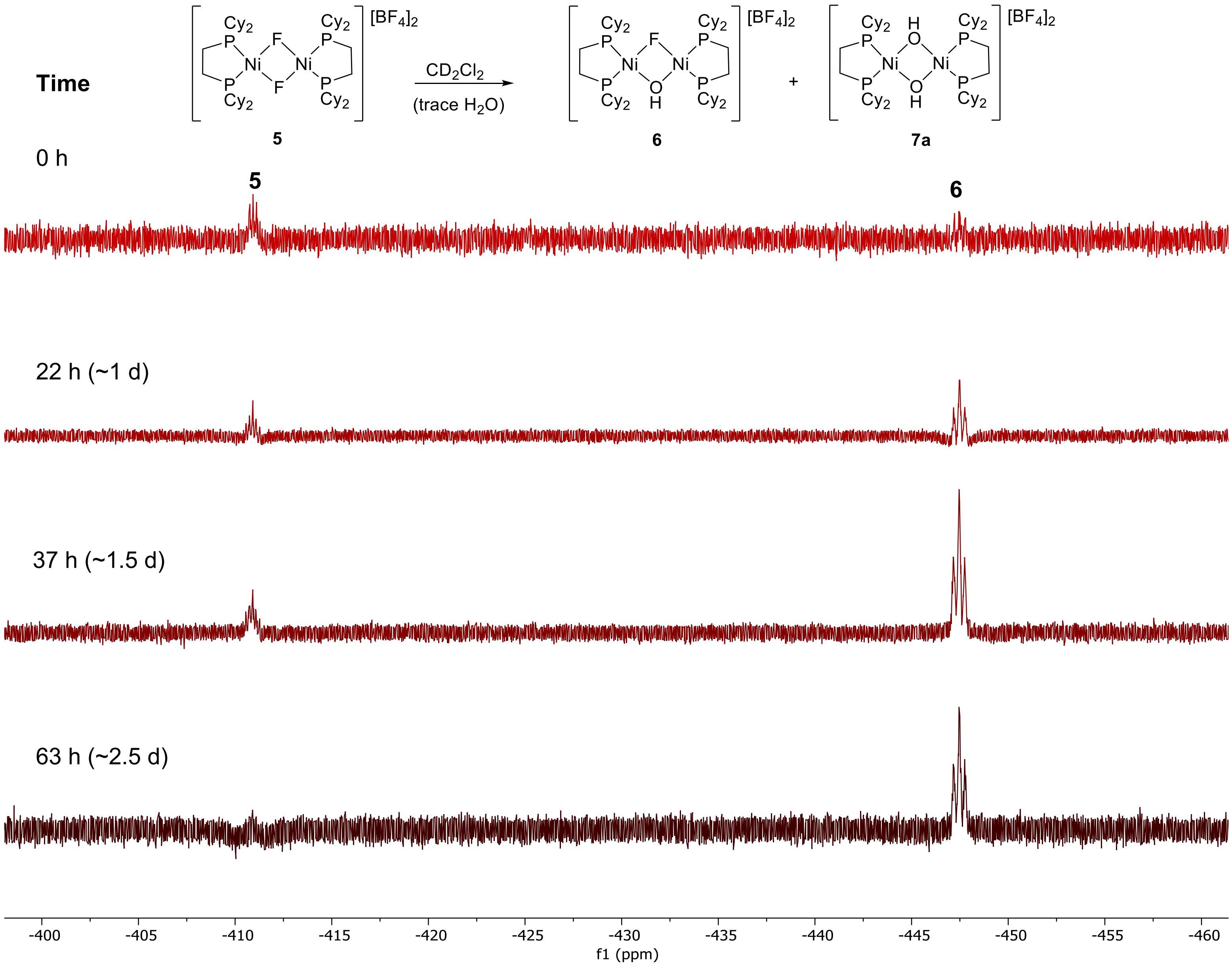

Figure S27 ^{1}H monitoring of the formation of the decomposition products of [(dcpe)Ni(μ-F)]$_2$[BF$_4$]$_2$ (**5**) dissolved in CD_2Cl_2 into [(dcpe)$_2$Ni$_2$(μ-F) (μ-OH)][BF$_4$]$_2$ (**6**) and [(dcpe)Ni(μ-OH)]$_2$[BF$_4$]$_2$ (**7a**) from 0 h to 2.5 d in an air-free glass NMR tube.

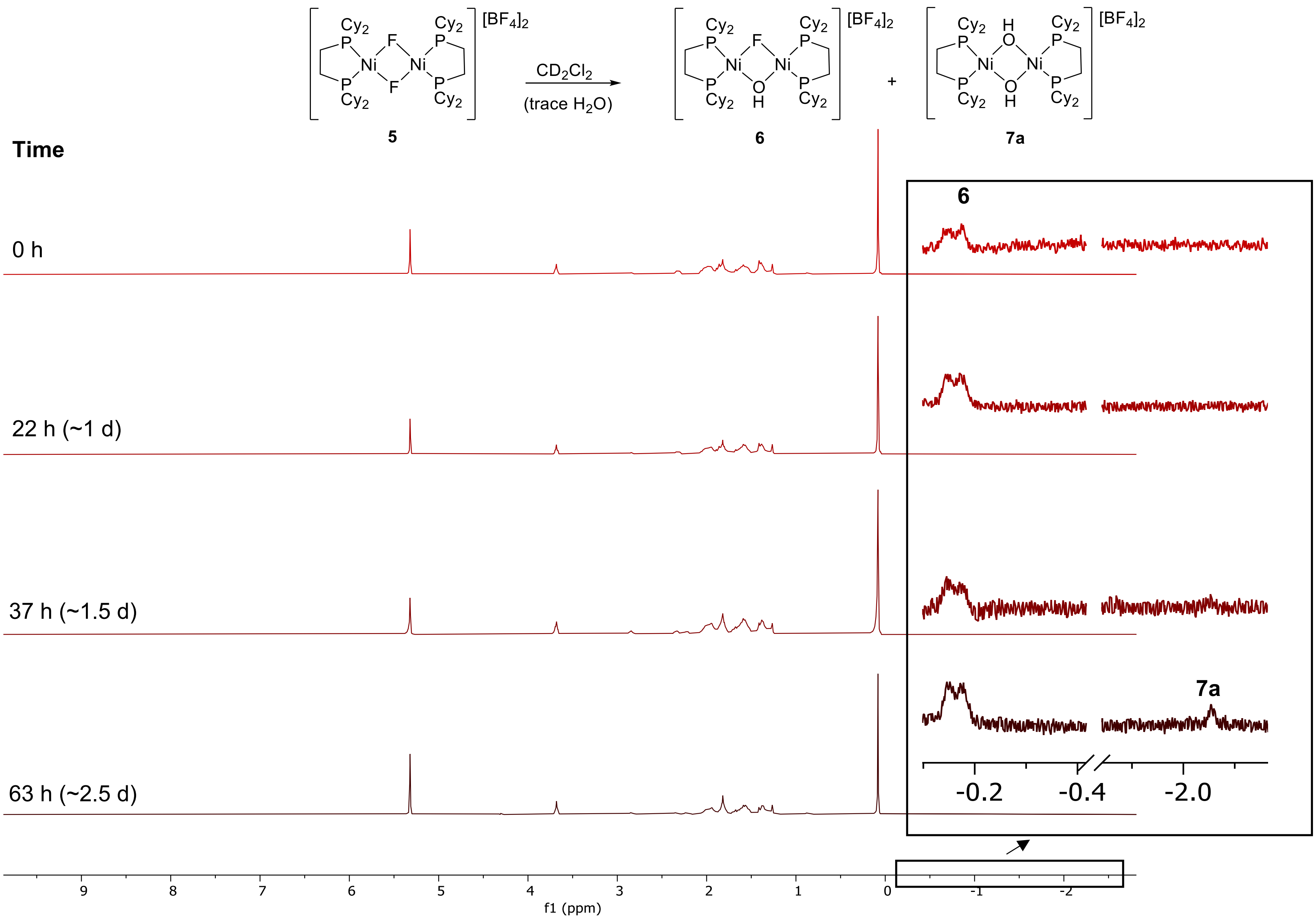

Figure S28 $^{31}P\{^{1}H\}$ monitoring of the formation of the decomposition products of [(dcpe)Ni(μ-F)]$_2$[BF$_4$]$_2$ (**5**) dissolved in CD_2Cl_2 into [(dcpe)$_2$Ni$_2$(μ-F) (μ-OH)][BF$_4$]$_2$ (**6**) and [(dcpe)Ni(μ-OH)]$_2$[BF$_4$]$_2$ (**7a**) from 0 h to 13 d in a PTFE-lined air-free NMR tube.

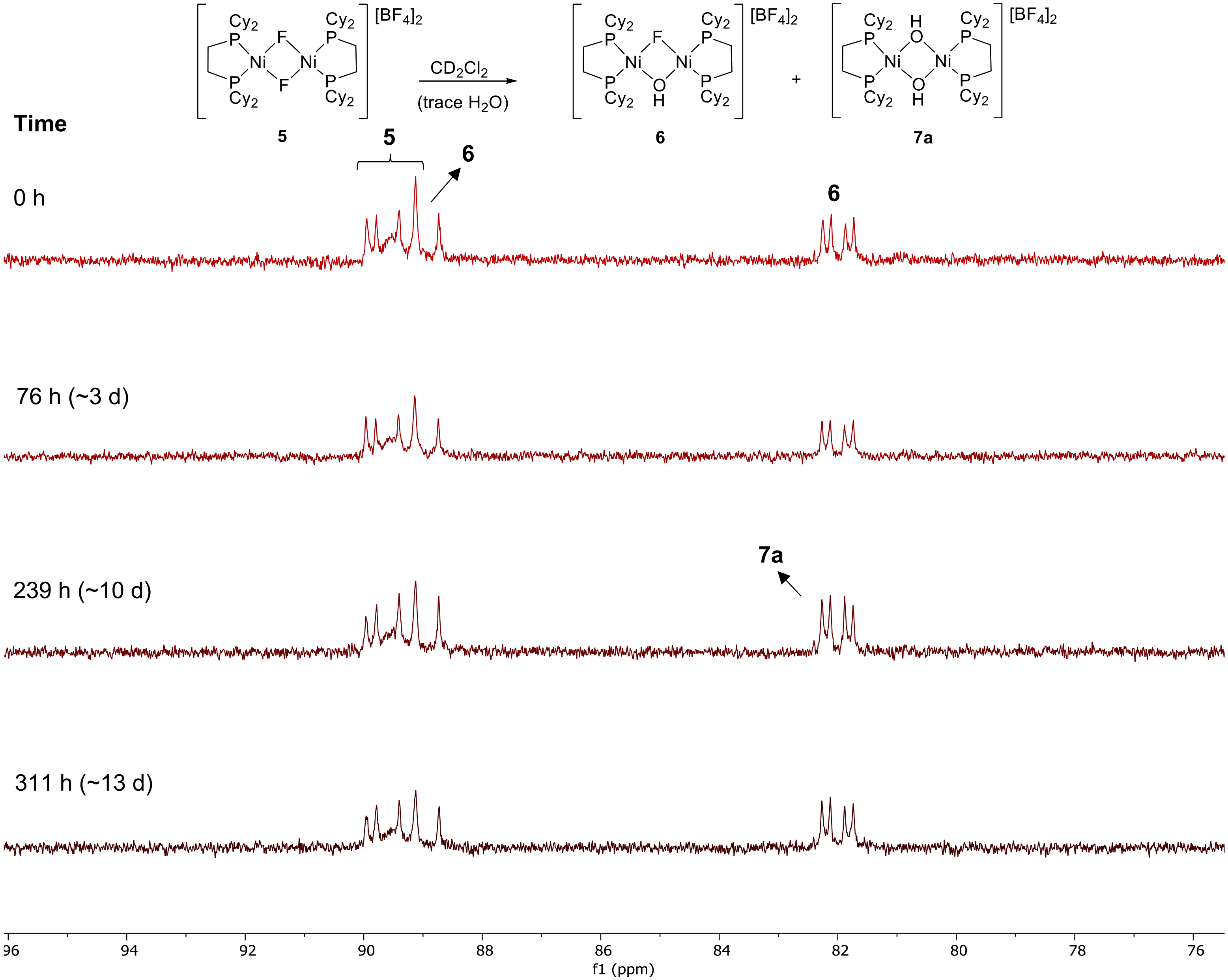

Figure S29 ^{19}F monitoring of the formation of the decomposition products of [(dcpe)Ni(μ-F)]$_2$[BF$_4$]$_2$ (**5**) dissolved in CD_2Cl_2 into [(dcpe)$_2$Ni$_2$(μ-F) (μ-OH)][BF$_4$]$_2$ (**6**) and [(dcpe)Ni(μ-OH)]$_2$[BF$_4$]$_2$ (**7a**) from 0 h to 13 d in a PTFE-lined air-free NMR tube.

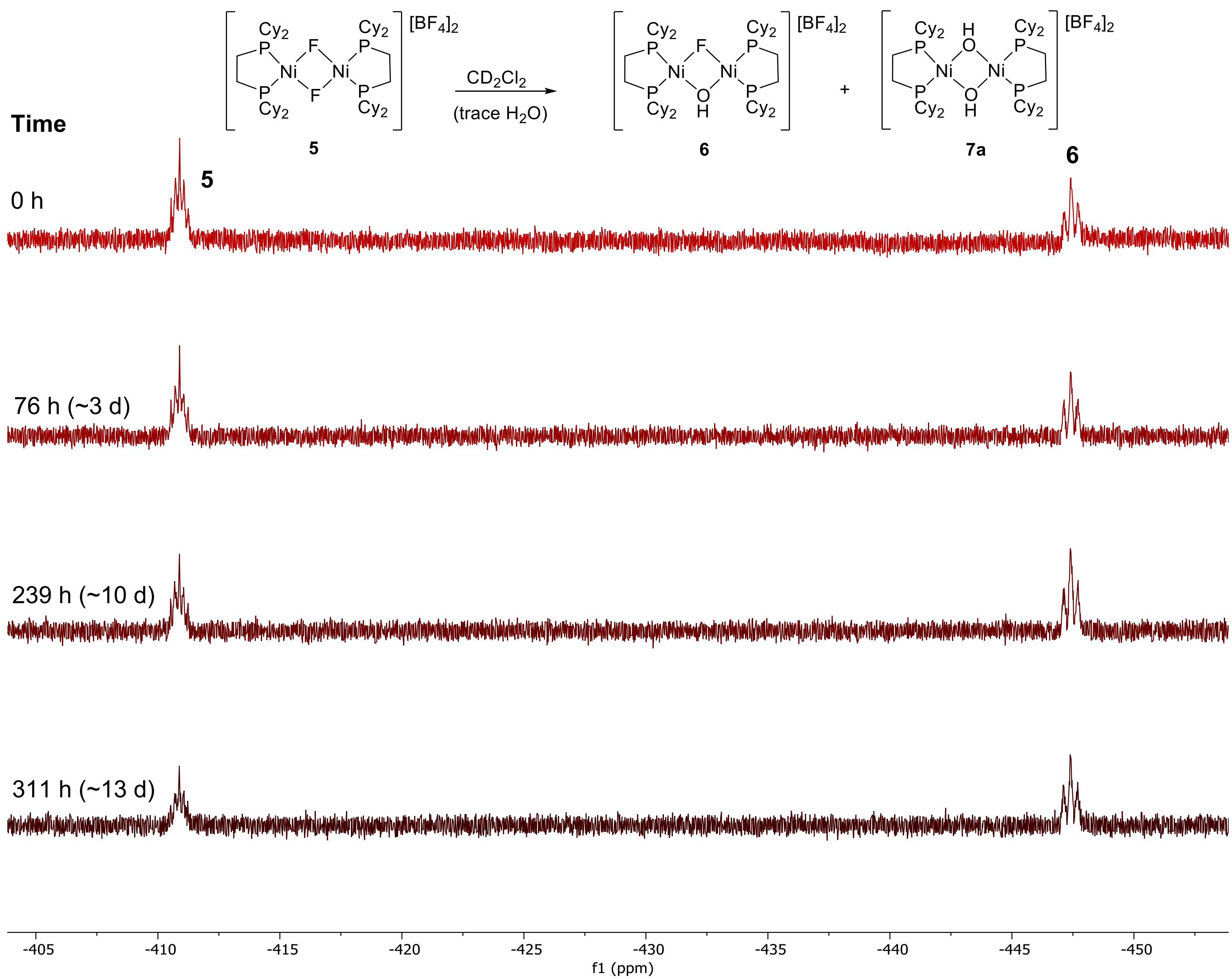

Figure S30 ^{1}H monitoring of the formation of the decomposition products of [(dcpe)Ni(μ-F)]$_2$[BF$_4$]$_2$ (**5**) dissolved in CD_2Cl_2 into [(dcpe)$_2$Ni$_2$(μ-F) (μ-OH)][BF$_4$]$_2$ (**6**) and [(dcpe)Ni(μ-OH)]$_2$[BF$_4$]$_2$ (**7a**) from 0 h to 13 d in a lined air-free NMR tube.

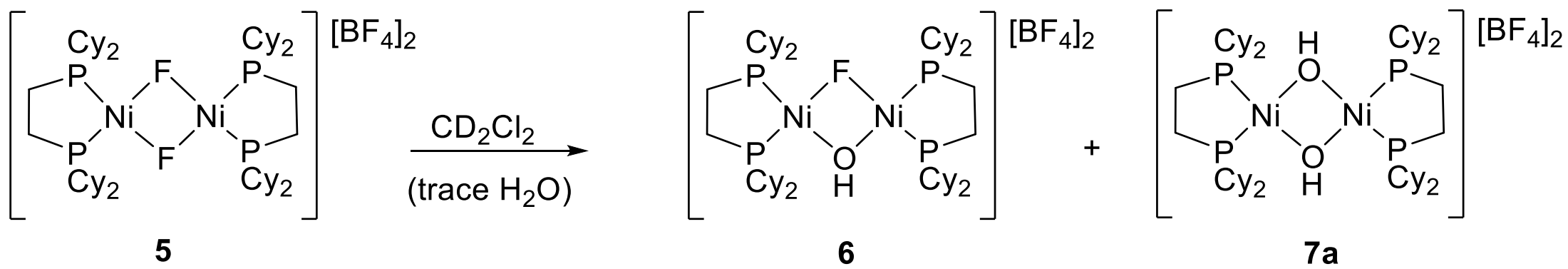

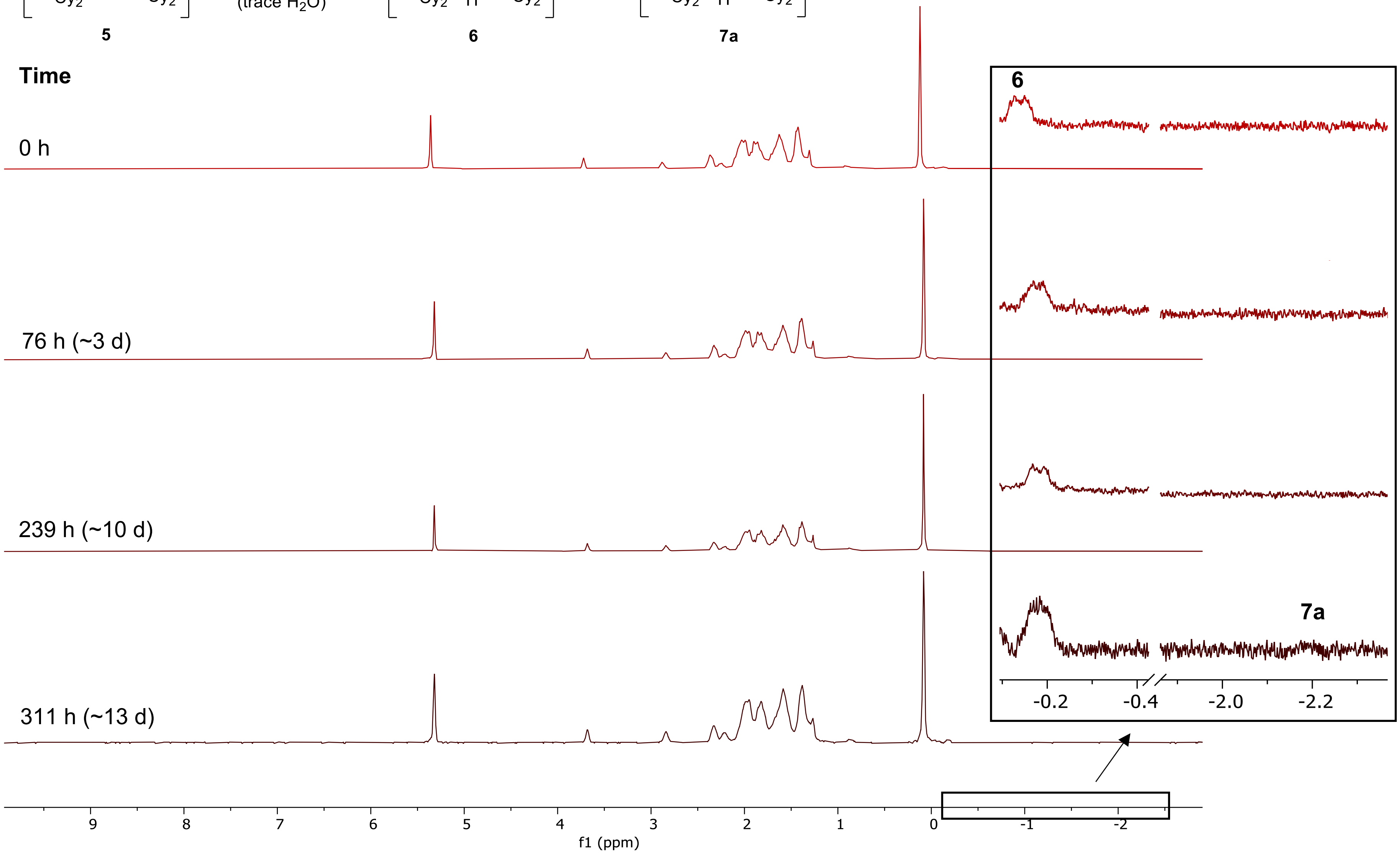

Figure S31 $^{31}P\{^{1}H\}$ spectra of the conversion of [(dcpe)Ni(μ-F)]$_2$[BF$_4$]$_2$ (**5**) to [(dcpe)Ni(μ-OH)]$_2$[BF$_4$]$_2$ (**7a**) and [NiCl$_2$(dcpe)$_2$] (**8**) when **5** is dissolved in undried CD$_2$Cl$_2$.

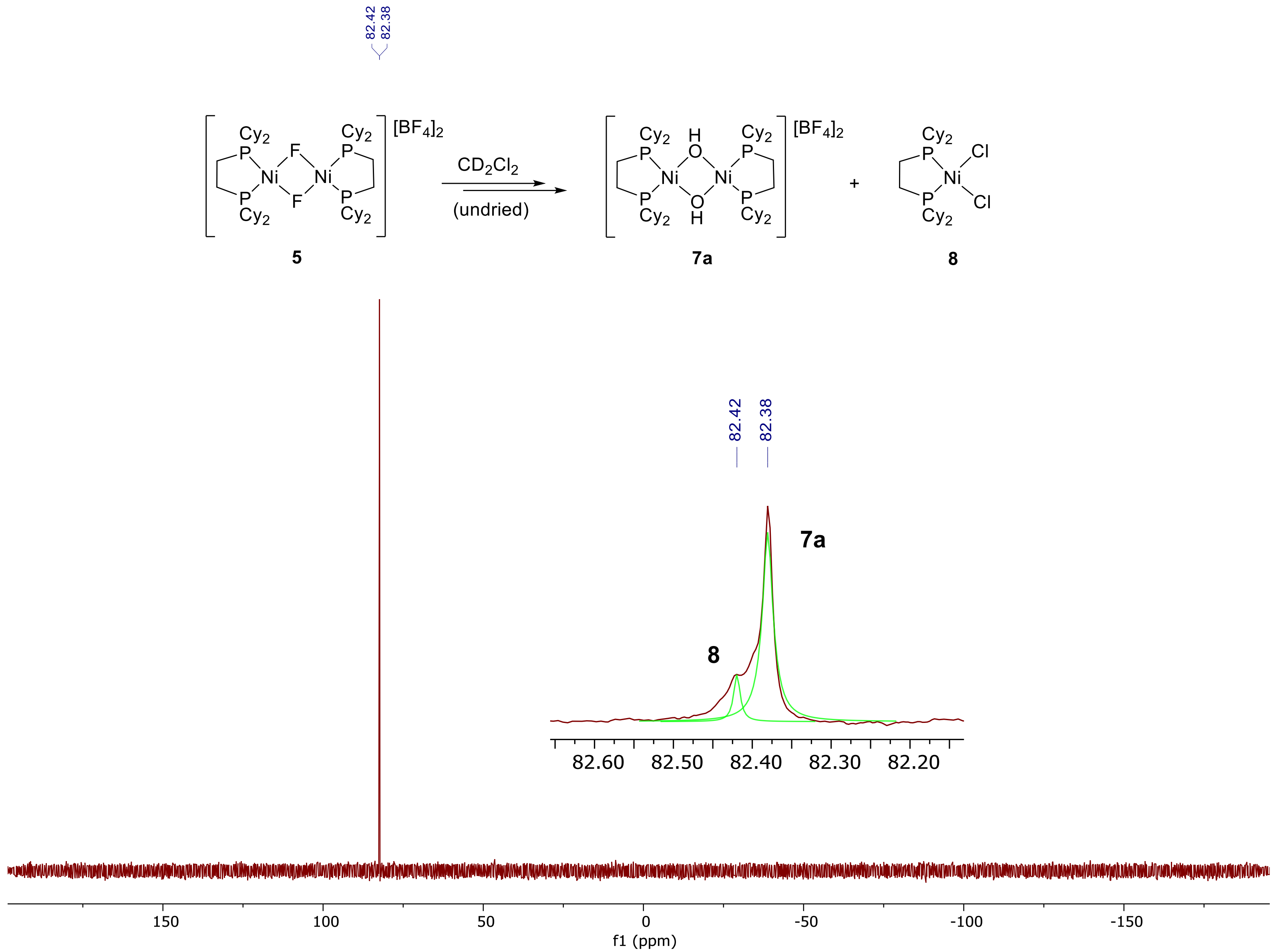

Figure S32 $^{31}P\{^1H\}$ spectra of the product formed from the reaction of $[(dcpe)Ni(\mu\text{-}OH)]_2[BF_4]_2$ (**7a**) with TMS-Cl to form $[NiCl_2(dcpe)_2]$ (**8**). This is a control experiment to differentiate **7a** from **8**.

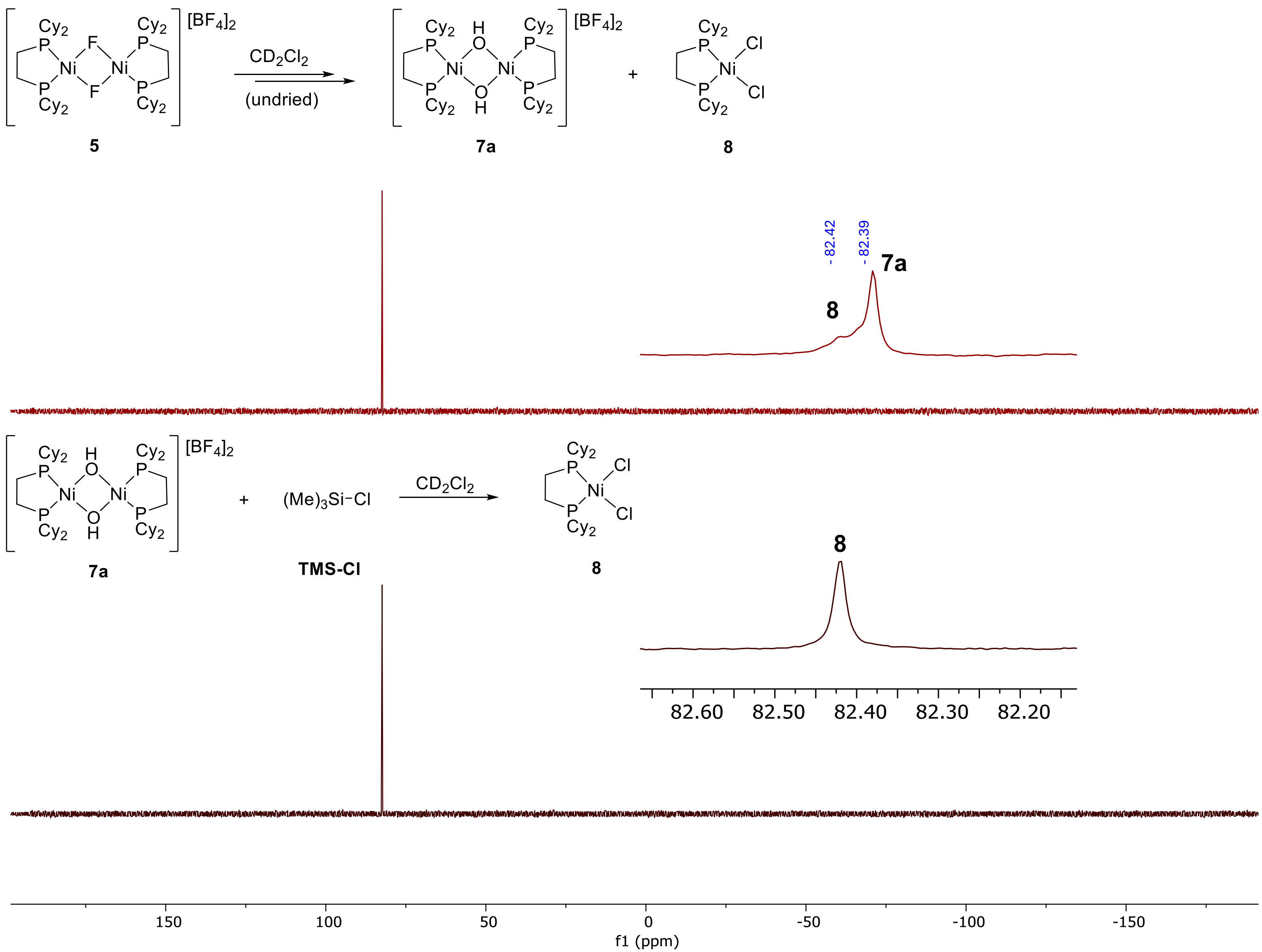

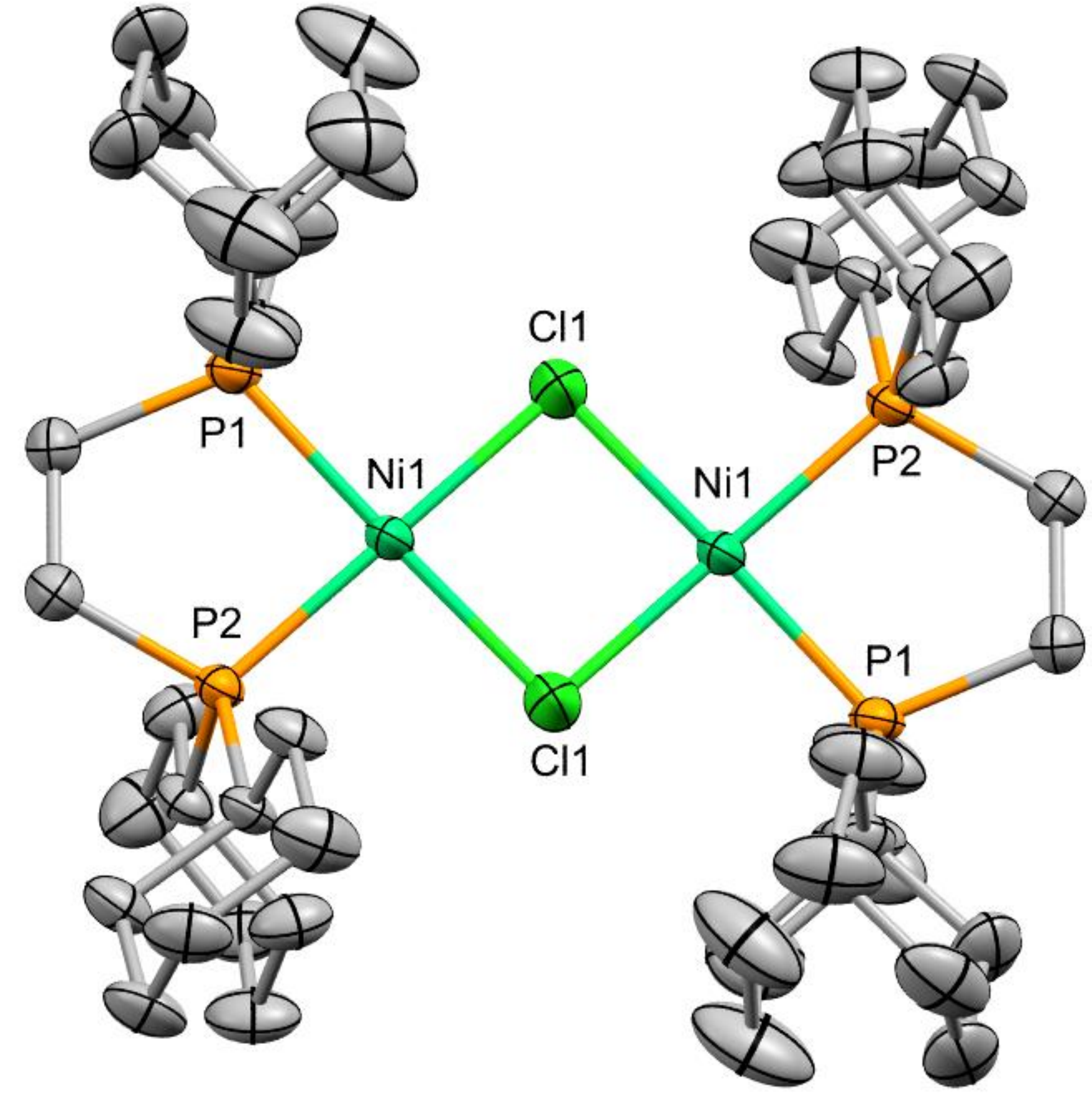

Figure S33. ORTEP diagram of the cationic core of [(dcpe)Ni(μ-Cl)]$_2$[BF$_4$]$_2$ **(4a)**. Hydrogen atoms, a co-crystallized THF molecule, and the BF_4 counter-anions are omitted for clarity. Thermal ellipsoids are shown at the 50% level. Selected bond lengths (Å) for **4a**: Ni1-P1 2.1545(12) ; Ni1-P2 2.1599(13) ; Ni1-Cl1 2.1973(15). Selected bond angles (°) for **4a**: P1-Ni1-P2 88.27(5); P1-Ni1-Cl1 173.24(7) ; P2-Ni1-Cl1 92.38(5); P1-Ni1-Cl1 91.42(5); P2-Ni1-Cl1 173.62(7); Cl1-Ni1-Cl1 88.69(6); Ni1-Cl1-Ni1 91.31(6).

References:

(1) Amel'chenkova, E. V.; Denisova, T. O.; Nefedov, S. E. Synthetic modeling of the active part of natural metalloenzymes by trimethylacetatozinc complexes. *Zh. Neorg. Khim.* **2006**, *51*, 1304–1348. DOI: 10.1134/S0036023606080110

(2) Hu, W.-Q.; Pan, S.; Xu, X.-H.; Vicic, D. A.; Qing, F.-L. Nickel-Mediated Trifluoromethylation of Phenol Derivatives by Aryl C-O Bond Activation. *Angew. Chem., Int. Ed.* **2020**, *59*, 16076–16082. DOI: 10.1002/anie.202004116.
